# Farming practices to enhance biodiversity across biomes: a systematic review

**DOI:** 10.1038/s44185-023-00034-2

**Published:** 2024-01-09

**Authors:** Felipe Cozim-Melges, Raimon Ripoll-Bosch, G. F. (Ciska) Veen, Philipp Oggiano, Felix J. J. A. Bianchi, Wim H. van der Putten, Hannah H. E. van Zanten

**Affiliations:** 1 Farming Systems Ecology Group, Wageningen University & Research, Wageningen, the Netherlands; 2 Netherlands Institute of Ecology (NIOO-KNAW), Wageningen Gelderland, Wageningen, Netherlands; 3 Animal Production Systems Group, Wageningen University & Research, Wageningen, the Netherlands; 4 Department of Food System Sciences, Research Institute of Organic Agriculture FiBL, Frick, Switzerland; 5 Laboratory of Nematology, Wageningen University and Research Centre, PO Box 8123, 6700 ES Wageningen, The Netherlands; 6 Department of Global Development, College of Agriculture and Life Sciences, and Cornell Atkinson Center for Sustainability, Cornell University, Ithaca, NY USA

**Keywords:** Ecology, Ecology, Environmental sciences, Agriculture

## Abstract

Intensive agriculture for food and feed production is a key driver of global biodiversity loss. It is generally assumed that more extensive practices are needed to reconcile food production with biodiversity conservation. In a literature review across biomes and for seven taxa, we retrieved 35 alternative practices (e.g. no-tillage, cover crops, organic fertilizer) from 331 studies. We found that no single practice enhanced all taxonomic groups, but that overall less intensive agricultural practices are beneficial to biodiversity. Nevertheless, often practices had no effects observed and very rarely contrasting impacts on aboveground versus belowground taxa. Species responses to practices were mostly consistent across biomes, except for fertilization. We conclude that alternative practices generally enhance biodiversity, but there is also variation in impacts depending on taxonomic group or type of practice. This suggests that a careful selection of practices is needed to secure biodiversity across taxa in future food systems worldwide.

## Introduction

Biodiversity is declining at an unprecedented pace around the world, with current estimates that around 5% of all species will be lost every ten years^1^. The main driver of biodiversity loss is agriculture^1^, both through intensification of existing agricultural land and expansion of agriculture into pristine ecosystems^2,3^. With a growing and wealthier population, food demand will further increase, accentuating the negative impacts on biodiversity^2,3^. The severe impact of agriculture on biodiversity stems from the intensification of agricultural practices in recent decades to increase yields to the detriment of other key ecosystem services, such as pollination, nitrogen cycling, carbon storage or resistance to drought^4^. Globally, these intensive practices, such as pesticide use, intensive tillage, and monocultures, have also been used in a similar fashion^3^ across biomes and climatic zones.

Management of agroecosystems aimed at the conservation and enhancement of biodiversity can play an important role in maintaining global biodiversity levels^5^. Therefore, a transition to more sustainable agriculture is being proposed, with multiple new systems emerging, such as circular agriculture, regenerative agriculture or ecological intensification, among others. These diverse systems put emphasis on distinct aspects of farming, such as soil management, use of resources or adoption of ecological principles. To achieve goals for sustainability in each of these systems, a range of management practices is used, with practices varying within and between systems, and potentially overlapping across systems. Therefore, agricultural practices are the most basic management unit of the farm system and can be referred to as the focal point of action. Often, the agricultural practices used in these emerging systems are less intensive than practices used in conventional agriculture (e.g., conservation biological control, minimum tillage). As a result, these alternative agricultural practices are acknowledged to improve biodiversity and the overall sustainability of agricultural systems^6–11^. However, earlier studies commonly focus on specific species/taxonomic groups in particular geographic regions or on particular combinations of practices but do not offer a global consensus on the impact of these practices on biodiversity around the world. While alternative practices often show positive impacts on specific species and taxa, it is unclear whether these practices can help maintain or enhance biodiversity levels of multiple taxa (i.e., pooling together all available taxonomic groups’ indicators for a given practice) across global agroecosystems. Ultimately, our agricultural systems need to be redesigned to enhance biodiversity.

In this study, we tested the hypothesis that alternative agricultural practices enhance agrobiodiversity when compared to intensive practices. To date, a comprehensive global overview of the impacts of intensive management practices and their less disruptive counterparts on a wide range of species and taxonomic groups is missing, as well as whether the effect of these agricultural practices is consistent across different biomes. Biodiversity in agroecosystems, henceforth referred to as “biodiversity”, is here comprised of different taxonomic groups (arthropods, birds, mammals, nematodes, earthworms, bacteria and fungi) representing both below and aboveground biodiversity. We aimed to evaluate synergies and contrasting effects of alternative practices on taxonomic groups.

Our review is the first road map of the literature identifying the impacts of agricultural practices on seven major species groups across biomes. We systematically reviewed 331 studies, resulting in 2538 data records representing unique combinations of settings (synchronicity of practices, location) and practices per species and/or taxa. We first (i) retrieved agricultural practices with documented impacts on biodiversity from the literature. We then (ii) assessed the qualitative impact (generally categorized as positive, negative or neutral) of each of these practices on each of the taxa in comparison to intensive practices. Lastly, we (iii) assessed whether the impacts of agricultural practices on biodiversity varied across biomes. Our findings show that no single practice enhances all taxonomic groups, but that alternative, less intensive agricultural practices are often beneficial to global biodiversity when compared to intensive conventional practices.

## Results

### Alternative agricultural practices

**Fig. 1:**
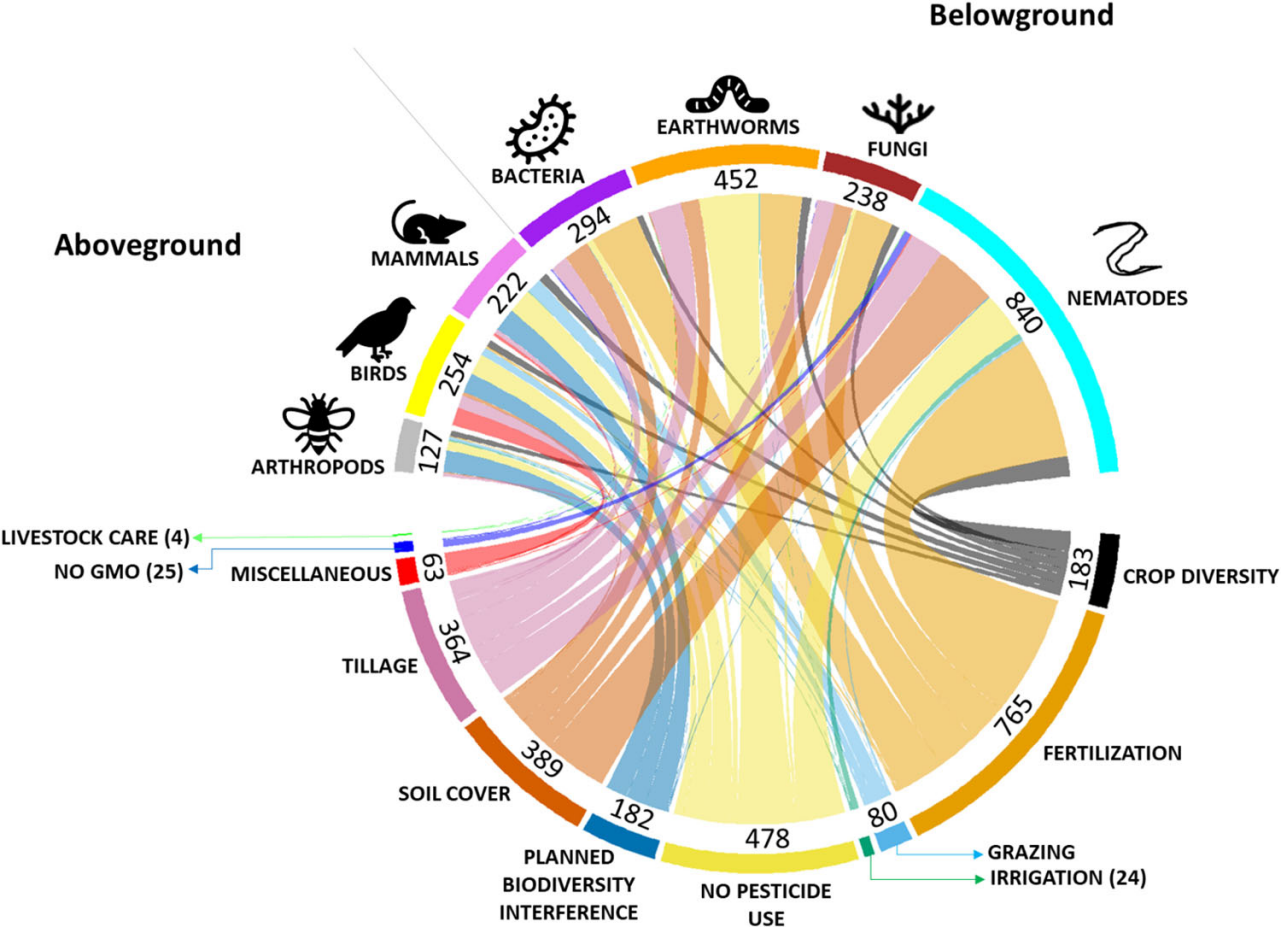
Overview of the distribution of reported impacts of groups of practices (bottom) per taxonomic group (top, represented by the same colour identity across all graphs and figures). Numbers indicate the number of data records in the literature review.

Our first objective was to identify what agricultural practices have been studied as alternatives to intensive ones. From the literature, we identified 35 alternative agricultural practices for which responses of seven studied taxa (arthropods, birds, mammals, earthworms, nematodes, bacteria and fungi) have been reported. We categorized these practices into ‘Groups of practices’ with a similar function (e.g., minimum tillage and conservation tillage) and compared them with their intensive counterpart practice (e.g., mouldboard tillage, referred to as intensive tillage). We then linked groups of practices with the taxa for which they were studied (Fig. 1). In total we identified eleven groups of practices: fertilization (containing five individual practices; *n* = 5); crop diversity (*n* = 2); planned biodiversity interferences (i.e., interventions either within fields or in surrounding areas aimed at enhancing biodiversity in the agricultural system, *n* = 2); no pesticide use (*n* = 3); no GMO use (*n* = 1); tillage (*n* = 4); soil cover (*n* = 5); irrigation (*n* = 5); grazing (*n* = 3); livestock care (*n* = 2) and others (referred to as “miscellaneous”, *n* = 3) (see Supplementary Material A for the definition of practices). In each group, we compare alternative practices to the commonly used conventional practice. For example, ‘zero tillage’, a practice where farmers abstain from tilling the land belongs to the tillage group and is compared to intensive tillage practices which we define as regular ploughing with full soil conversion and leaving <30% crop residue cover. Data records from the literature review were assigned to individual practices within the groups and were classified as positive, negative or neutral for each studied taxonomic group. We found that practices are studied heterogeneously across taxa and while some practices are well studied (e.g. fertilization) or include many taxonomic groups (e.g., crop diversity), this is not the case for other practices (e.g., planned biodiversity interference or grazing, Fig. 1). We also observed strong inclination of ‘planned biodiversity interferences’ to targeting aboveground biodiversity, while fertilization was mostly studied for belowground biodiversity. ‘No pesticide use’ was the most broadly studied group across taxa.

### The impact of alternative practices on biodiversity

**Fig. 2:**
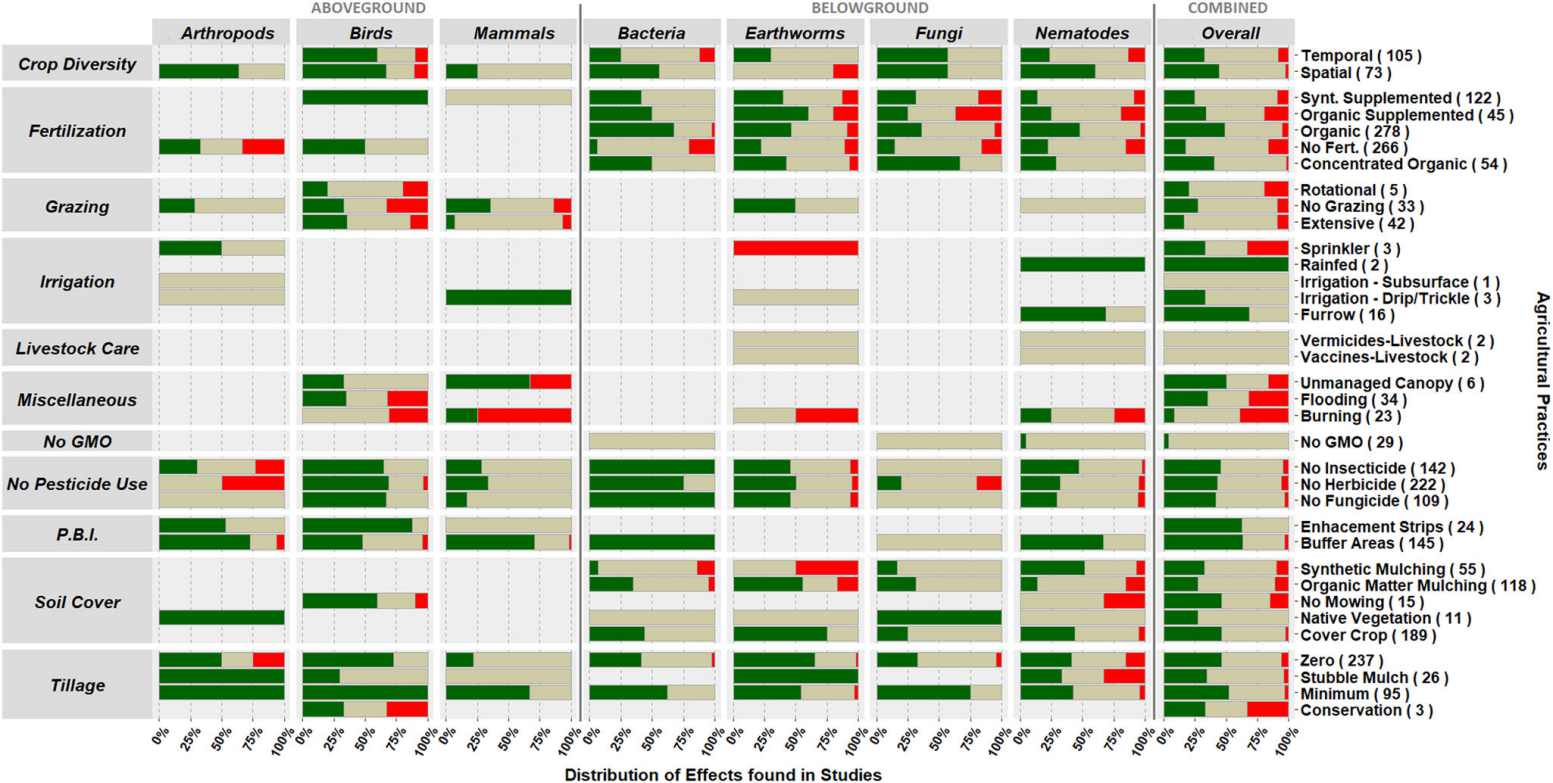
Responses of seven taxonomic groups of above- (arthropods, birds and mammals) and belowground biodiversity (bacteria, earthworms, fungi and nematodes) to alternative management practices relative to intensive mainstream practices defined in Supplementary Material A. Numbers in parenthesis indicate the number of data records per practice. Bars indicate the distribution of cases in which alternative practices lead to positive (green) or negative (red) when effects were significant in the studies from which they were retrieved, or neutral (beige) when no significant effects were observed on the taxa studied for the combination of all biodiversity metrics retrieved. The practices of livestock vaccines, livestock-vermicides and irrigation subsurface were not included due to scarcity of data. The number of studies and data records per practice/taxa can be found in Supplementary Material F. P.B.I. refers to ‘Planned biodiversity interferences’.

When compared to intensive practices, the majority of groups of alternative practices enhanced or were majorly positive to biodiversity, with a large incidence of no observed effects as well (Fig. 2). Although studies have used diversity, species richness and abundance, the majority focused on abundance, with aboveground taxa having a larger share of richness and diversity metrics, and effects were often consistent across the different indices (Supplementary Material B). Consistent positive responses on combined available taxa, henceforth referred to as overall biodiversity, were found for all practices belonging to ‘planned biodiversity interferences’ and “no pesticide use”, (Fig. 2, Supplementary Material B). However, specific practices within the same ‘group of practices’ sometimes impacted overall biodiversity differently. For instance, ‘no fertilizer’ affected biodiversity more negatively than ‘bulky organic fertilizers’. Our results show that although most alternative practices are more biodiversity-friendly than intensive practices, some alternatives are less benign or might very often have no observed impact on some or as many taxonomic groups as they have positive effects (i.e., no fertilizer). This becomes clear when we focus on the impact of individual practices on individual taxonomic groups instead of overall biodiversity (Fig. 2, Supplementary Material B).

Zooming-in on individual practices and individual taxonomic groups we observe that individual practices affect individual taxonomic groups differently (Fig. 2). For example, the practices within the group ‘no pesticides’ seem to have a positive impact on most taxonomic groups (Fig. 2), but not on fungi. Most practices had majorly positive effects, some 100% or nearly 100% positive, however, these cases were usually specific to practices with few data records, such as “unproductive biodiversity zones” for birds (8) and “native grass vegetation” for fungi (1). Nevertheless, some specific practices also had both a majorly positive proportion of effects to specific taxa and a large amount of data records corroborating these results (*n* > 25). Arthropods and mammals had consistent positive responses to ‘natural buffer areas’ (*n* = 33 and 61, respectively) while birds were mostly enhanced by ‘no herbicides’ (*n* = 26). Earthworms were the only taxa consistently enhanced by two practices, ‘zero tillage’ (*n* = 55) and by the use of green/living ground cover (‘cover crops’, *n* = 28). Bacteria, on the other hand, only had consistent positive responses to the use of ‘organic fertilizer’ while the biodiversity of fungi and nematodes were not positively affected in a consistent way by any of the groups of practices, despite having most effects of alternative agricultural practices being positive.

The evaluation of the impacts of individual practices on individual taxonomic groups becomes challenging when taxonomic groups show group-specific responses to practices. We have observed many instances where positive effects were observed for certain taxa while mostly no observed effects were observed for other taxa, e.g. ‘no pesticide use’ on mammals and fungi. At rare occasions contrasting effects of practices were observed between taxonomic groups, i.e. ‘no mowing’, ‘synthetic mulching’, ‘spatial crop diversity’, ‘organic fertilizer’ and ‘no fertilizer’. For example, ‘synthetic mulching’ seemed to benefit fungi and nematodes, while adversely affecting bacteria and earthworms. Furthermore, practices can have contrasting impacts on overall above- and below-ground biodiversity. In the rare cases where these contrasting effects were observed, practices enhanced aboveground biodiversity and reduced belowground biodiversity. Earthworms were part of three of the five cases where potential contrasting effects occurred, indicating that earthworms are a sensitive species group.

To summarize, we identified four practices that benefitted multiple taxonomic groups, henceforth referred to as “no regret” practices, that enhance combined biodiversity without adversely affecting any specific taxonomic group, i.e. practices in the group of ‘planned biodiversity interferences’ and ‘no fungicide’ and ‘no insecticide’. We also found five individual practices that enhanced specific taxa, i.e., the use of buffer areas (arthropods and mammals), the absence of herbicides (birds), no-tillage (earthworms), organic fertilizer (bacteria), and cover crops (earthworms), referred to as “targeted” solutions. We found that applying those “targeted ” individual practices has the potential to consistently positively affect the agroecosystem biodiversity of their target taxa. We show that these individual practices are capable of enhancing five of the seven taxa studied (i.e., arthropods, birds, mammals, earthworms and bacteria).

### Consistency of the impact of practices across biomes

**Fig. 3:**
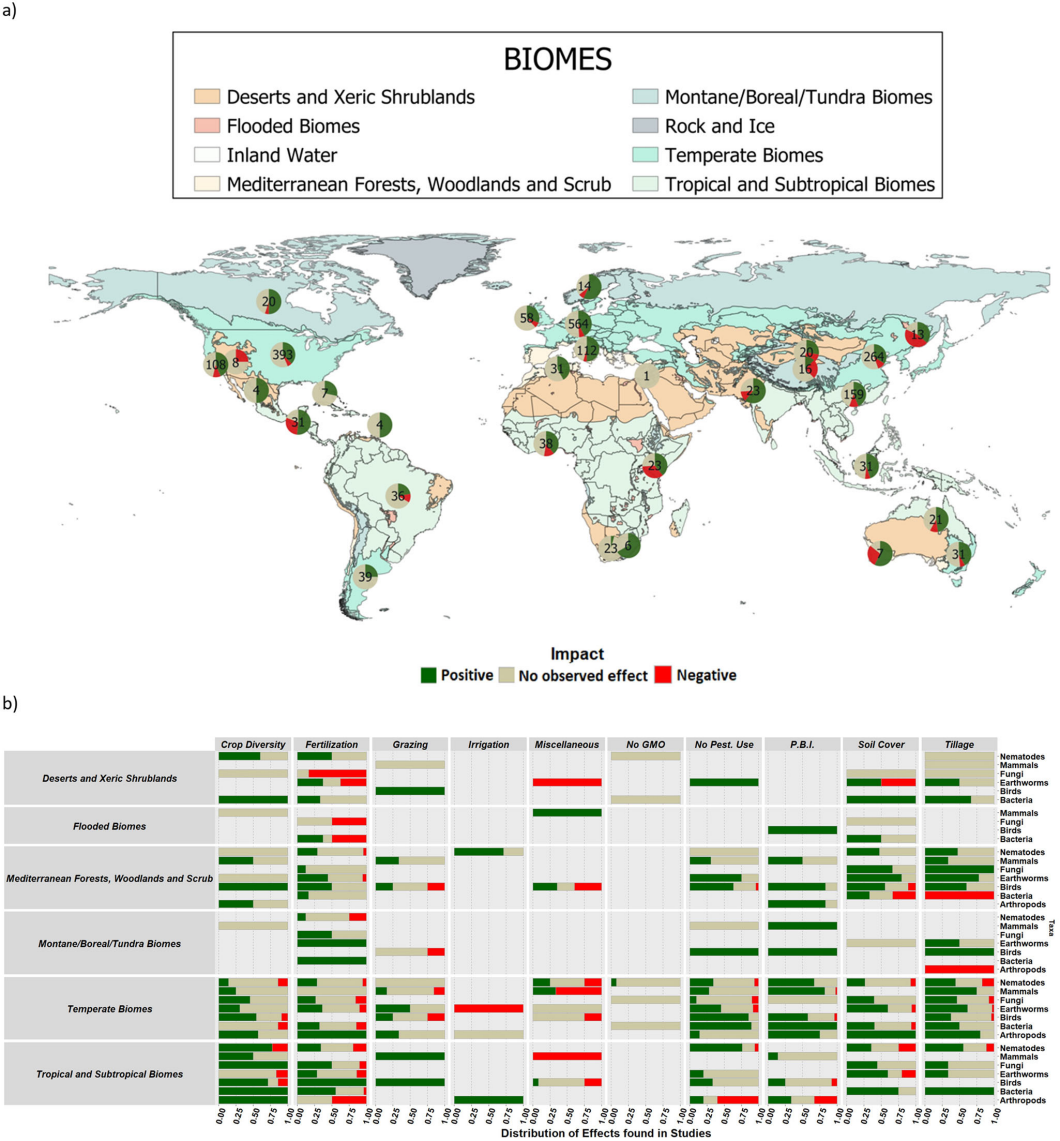
The distribution of data records in biomes in the globe and the proportion of positive, negative and neutral effects of the groups of practices on taxa in each of the biomes. **a** The distribution of data records across the globe and the biomes they are located clustered based on their ecoregion parcel in each region/continent, e.g. Germany, France and Hungary data records clustered in Europe. Green colour represents positive effects, red represents negative effects and beige represents neutral effects (when no significant effects were observed in either direction in the studies from which they were retrieved). **b** The proportion of effects of each group of practices across taxa for each of the biomes studied. ‘P.B.I.’ refers to the group practice ‘planned biodiversity interferences’.

The effects of groups of practices on taxonomic groups were mostly consistent among six biomes. The exceptions were alternative fertilization practices which had solely positive impacts on earthworms but had majorly negative effects for arthropods in tropical and subtropical biomes, fungi in deserts/xeric shrubland, bacteria in flooded biomes and a mostly neutral, but negatively inclined distribution for nematodes in montane/boreal/tundra biomes (Fig. 3). However, the proportion with which groups of agricultural practices impact taxonomic groups varied across biomes. Observations were mostly distributed similarly in terms of the proportion of effects across all biomes for each taxonomic group (Fig. 3b), with the following exceptions: ‘crop diversity’ for mammals, birds and arthropods; ‘grazing’ for mammals and birds; ‘fertilization’ for fungi and earthworms; and ‘tillage’ for bacteria. Fertilization and tillage had biome-specific variations from positive to negative on the effects for fungi, bacteria, nematodes and arthropods. However, fertilization was the only group of practices where the impact varied from positive to negative for different biomes for four taxa, i.e. nematodes, bacteria and fungi, as well as arthropods. However, these variable impacts may partly be explained by the relatively low sample size of biomes different than temperate and Mediterranean. Nearly 64% of the observations (1475 data records) originated from temperate biomes, 16% (365 data records) from tropical biomes, and 14% (333 data records) from Mediterranean biomes. All other biomes combined represented the remaining 6%, highlighting the uneven availability of research data across biomes. Furthermore, the variation within biomes is largely driven by specific combinations of taxonomic groups and groups of practices, e.g., tillage has a much wider variation in the proportion of effects across biomes for bacteria than it does for earthworms.

## Discussion

In line with our hypothesis, this review shows that most alternative practices have positive effects on biodiversity in agroecosystems. However, this is not the case for all individual practices and/or for the effects on all taxonomic groups, with many of them having mostly no observed effects. Many previous reviews or meta-analyses on the impacts of farming on biodiversity have either focused on farming systems as a whole^6,9^, such as circular agriculture^12^, regenerative agriculture^13^ or organic agriculture^14^, or on individual practices or taxonomic groups^15–17^. As a result, these reviews did not systematically assess the effects of shifts in a range of agricultural practices across a range of different taxonomic groups (e.g., grazing^18,19^). Our study better represents the impact of these alternative practices on overall biodiversity and helps bridge the gap between reconciliating agricultural practices and biodiversity, represented by a wide range of taxonomic groups, both above and belowground, or as combined biodiversity. Our results show that there are differences in how and which certain practices positively impact certain taxa, especially when these impacts are mostly consistent across biomes. In the following sections, we discuss this in more detail by focusing on (i) the practices identified; (ii) the impact of each practice on biodiversity, overall and for individual taxonomic groups; and (iii) the relevance of biomes in the impact of groups of practices on taxonomic groups.

We identified clear biases and gaps when linking practices to taxonomic groups, as certain practices were only studied for specific taxonomic groups. For example, the impact of fertilization was mainly studied for belowground biodiversity, while, in contrast, studies for natural buffer areas mainly focused on aboveground taxa. While this above and belowground focus follows intuitively from the nature, aim and scale of the practices^20–23^, it prohibits assessing the impacts of many of the practices on biodiversity across above and belowground and its multiple taxa^24^. Therefore, it is crucial to start integrating knowledge on the impacts of agricultural practices on biodiversity across taxa^25^ because specific taxa may respond differently to a particular practice^23^. Our findings are in line with Billeter et al. ^26^, who argued that using particular, single taxa as indicators for biodiversity does not necessarily represent biodiversity as a whole and can therefore be misleading.

As to the impact of practices, in line with our hypothesis, we found that most alternative agricultural practices enhanced overall biodiversity. Practices in the group of planned biodiversity interferences and no pesticide use were clearly positive in a vast majority of cases, with almost no negative incidence across taxonomic groups. The finding that alternative practices benefit biodiversity is coherent with earlier work showing that some of these practices^16,19^ and associated agricultural systems^6,9^ gave positive outcomes for biodiversity. These positive impacts support the expectations noted by Newbold et al. ^27^, who argued that more extensive practices could be more positive for biodiversity. This is indicated by our results, where less intensive tillage, for example, reduced the disturbance of the earthworm environment^28,29^. The overarching implication from these results is that biodiversity profits from the extensification of practices. Nevertheless, biodiversity-friendly practices alone will not suffice and should be coupled with a concomitant strategy to prevent further expansion of agricultural lands and restore and preserve existing natural areas^30–32^.

We were able to determine the impacts of types/categories of practices on biodiversity. However, it is expected that the intensity with which alternative practices are applied, e.g., the amount of fertilizer applied, will influence how biodiversity responds to changes in agricultural management. Our assessment required a broad and encompassing methodology, hence a qualitative approach was used. The trade-off with our method is that it does not allow for determining the magnitude of the effect of the practices^33–36^ but only the proportion of impacts^33,37^ as positive, negative or neutral. Moreover, biodiversity impacts of practices may vary over time, such as the seasonality effect on species, and duration of the practice impact (Press-pulse dynamics^38,39^). These aspects, however, were beyond the scope of this review. Therefore, to assess the biodiversity impacts of practices in the future, not only the intensity of practices needs to be considered, but also the timing, frequency and duration of these practices^40^. In this way, practices can be better applied to enhance biodiversity accounting for the complexity of the field and landscape management. The findings demonstrate that practices in the group of ‘planned biodiversity interferences’ and ‘no pesticide’ yield positive outcomes, exhibiting minimal adverse effects across taxonomic categories. Hence, these practices seem to be immediate options to be implemented to enhance biodiversity.

Most practices, however, were either not studied for all taxa included in this review or only affected some of the taxonomic groups. These results may be partly driven by gaps in the literature (i.e., some practices were not studied for some groups). Therefore, it will be essential to understand the impact of practices across multiple taxa, because our finding indicated that responses of different taxa to practices were variable, sometimes even opposite. For instance, aboveground biodiversity was strongly enhanced by the presence of natural buffer areas, while fertilization and tillage mostly enhanced belowground biodiversity. Even the practices with substantially positive biodiversity outcomes, such as no pesticides, showed limitations on improving particular taxa such as fungi. Furthermore, despite mostly positive effects, we were unable to identify practices that significantly enhanced the biodiversity of fungi and nematodes. This is in contrast to Morugan-Corunado et al. ^16^, who found that reduced tillage enhanced the abundance and diversity of fungi due to a reduction in soil disturbance. This variation and contrasts in responses may be partly caused by variation in biodiversity indicators across studies e.g., diversity, richness and abundance, with aboveground studies generally focussing on diversity and richness and belowground ones on abundance. In addition, cases with contrasting impacts for different taxa mostly involved practices with few data records (e.g., irrigation sprinkler, no mowing, synthetic mulching) or very few negative effects(e.g., organic mulching, organic supplemented). While these contrasting effects were not frequent and distributions involved smaller sample sizes, the findings signal that practices might benefit some taxa while adversely affecting others, and need to be considered and further studied. For instance, while mulching is positive for most belowground biodiversity, synthetic mulching seems to adversely affect earthworms, a taxon responding substantially positively to other practices in that group. If we disregard these contrasting effects we might be applying a practice to enhance biodiversity but adversely affecting other taxa. Interestingly, all cases of contrasting responses between above and belowground taxa involved negative impacts on belowground taxa and positive effects on aboveground taxa, which highlights a potential difficulty in applying some of these practices to enhance biodiversity in above and belowground communities at the same time. Whether these responses indicate a trade-off between the presence of certain taxa or, rather, opposing effects to the same practice between above and belowground compartments, needs further investigation^41^.

Our findings imply that the evaluation of the impact of practices varies depending on the level of aggregation of the biodiversity assessment (i.e., whether the impact is assessed at the species level, taxonomic group or above/belowground compartment). Yet, we only retrieve the impacts as qualitative data, and understanding the magnitude of the impacts of different practices on biodiversity across taxa will be essential to make further suggestions for agricultural management. This was not possible in our review, as one of our key aims was to also retrieve what practices are studied and for which of the seven taxa. Therefore, our results now provide a roadmap of the literature studying the impact on biodiversity of agricultural practices. A key next step is to analyse specific individual practices in-depth using meta-analyses techniques. Also, it will be essential to move beyond the richness and abundance of taxonomic groups, integrate functional or trophic aspects of biodiversty^42,43^ and community composition^44–46^. In addition to identifying no-regret practices that positively affect overall biodiversity, we were able to find different practices that frequently positively affected specific taxa, while not adversely affecting others. These ”targeted” practices may represent a promising solution for enhancing biodiversity for specific taxa. Our results indicate that general enhancement of multiple taxonomic groups could be achieved through the application of a combination of both no-regret and targeted practices. Furthermore, there may be synergies or disruptions among these practices (e.g., tillage regime and fertilization type), so to truly understand which practices should be combined it will be essential to study these interactions.

In regards to the relevance of biomes, and supporting our hypothesis that alternative practices would enhance biodiversity, we found that the distribution of impacts of agricultural practices was mostly consistent across biomes in terms of their direction. However, the proportion of the direction of impacts differed between biomes for some practices, e.g., pesticide use and tillage. Belowground biodiversity varied more in terms of the direction of impact of practices, but aboveground taxa had a wider range of proportion of impacts for the same group of practices across biomes. Whether soil type (or the combination biome/soil), rather than biome is a better determinant of how practices affect belowground biodiversity^47^ is a current topic of debate. However, while we found no results supporting biomes changing the impact of practices, Morugan-Coronado et al. ^16^ found no evidence of soils affecting the impact of practices either. Our findings suggest that alternative practices are more biodiversity-friendly than mainstream intensive practices, irrespective of the biome where it is implemented. However, there were considerable data gaps in terms of whether all biomes possessed all targeted taxa and practices. For instance, tillage was only studied for four of the seven taxa for tropical and subtropical biomes. Additionally, the clear bias in the biomes stresses the importance of improving efforts to collect data from these regions where most of the biodiversity conservation potential resides^48^. Further studies in the global south could provide a better understanding of the impacts on different biomes.

Agroecosystems should play a key role in the effort to conserve global biodiversity^5^. Referring to our hypothesis, we conclude that alternative agricultural practices can enhance biodiversity, but not all practices affect biodiversity in the same manner. Enhancing biodiversity requires both practices that improve combined biodiversity conditions, but also targeted practices that enhance specific species while not adversely affecting others. Hence, rather than focusing on a specific framework with which to redesign farming systems (e.g. circular agriculture, regenerative agriculture, or ecological intensification), we argue that focusing on practices is the starting point to recovering biodiversity in agroecosystems. First, because some of the practices here reviewed can already be implemented in current agricultural systems to deliver positive outcomes on biodiversity (this review)and underlay and overlap across many of the proposed frameworks. Hence, adopting practices instead of frameworks sounds more easily attainable. In any case, we understand that agricultural practices are de facto ecological disturbances and hence, further expansion of agricultural lands into natural areas should be avoided. With this research, however, we suggest that in areas where simplified, intensive agricultural systems have been the norm, alternative practices may help to create more complex agroecosystems that benefit biodiversity. Thus, we believe that the aim of agroecosystems should be to enhance their biodiversity instead of simply preserving it. More biodiverse systems will present benefits such as higher resilience^49–51^ and a wider range of ecosystem services^52–55^. Biodiversity conservation is a systemic issue and our results clearly stress the importance of simultaneously addressing multiple aspects of biodiversity (e.g., soil organisms, arthropods, birds) for effective conservation.

## Methods

### Agricultural practices

Agricultural practices in this study were retrieved as an outcome of the literature review; whenever an agricultural practice was found in more than one study it was added to the list of agricultural practices. To relate the practices to their function, we clustered them in broad groups describing the nature of the agricultural practices, henceforth termed “group of practices”. These groups of practices clustered the individual practices into types of practices, such as Fertilization (synthetic, bulky organic application), tillage (intensive tillage, conservation tillage, no-tillage) and no pesticide use (no herbicide use). The practices found often varied in their application and specificity from study to study and even in how they were assessed for each taxon. Therefore once a practice was retrieved we merged their entries based on and into the common definition, as per the description of the practice in supplementary Material A, (e.g., organic pellets and slurry application as ‘concentrated’). These common definitions and groups of practices allowed identifying the intensive agricultural practice, which was used as a reference to compare to the alternative practice (e.g., ‘zero tillage’ was in the group ‘tillage’, which had as intensive reference ‘intensive tillage’) and determine the biodiversity impact. Due to the breadth of the literature review, we also found practices with specific functions and applications. These did not necessarily have an intensive control group, but were still found in multiple studies and hence conformed with our criteria. In order to maintain a comprehensive overview based on practices studied for the targeted taxa, we clustered them in a group of practices called ‘miscellaneous’, representing all practices with specific and context-dependent applications. Therefore, the ‘miscellaneous’ group of practices was the one exception where no single intensive practice was used for all of them due to their nature. The retrieved practices in this literature review, referred to as alternative practices, were classified using broad practice definitions to attend to the specific practice conditions of the geography it was applied. All practices used in this review as well as their respective groups, intensive practice control, and respective definitions can be found in Supplementary Material A.

### Biodiversity indicators and targeted species

To represent biodiversity in agroecosystems, this study focused on major above and belowground taxonomic groups, including birds, mammals, arthropods, nematodes, earthworms and soil microorganisms (i.e. fungi and bacteria). To translate these concepts into the literature review, some taxa, such as arthropods, were divided into target species relevant to agroecosystems (Table 1). Target species were chosen to be used in the search strings based on their relevance in different niches and trophic levels to agroecosystems as found in the literature. In the case of arthropods, bees and wasps were chosen, and in the case of mammals, small rodents and bats. This criterion was based on their pollination and biocontrol potential, as well as their documented presence in such systems around the globe. For birds, no target species were chosen. Soil biodiversity in the form of nematodes, earthworms, bacteria and fungi had well-established functional groups in the literature. A list of all species and their corresponding indicators and data points can be found in Supplementary Material B.

**Table 1:** Target groups of the search queries and their respective species

Taxa	Target	Initial literature basis
Arthropods	Bees, Wasps	Kennedy et al.^9^, Bengtsson et al.^6^
Mammals	Bats, Rodents	Williams-Guillen et al.^58^, Coda et al.^59^
Birds	General	Bengtsson et al.^6^
Nematodes	Herbivores, Bacterivores, Fungivores, Omnivores-Predators, Entomopathogenic	Yeates^60^
Earthworms	Epigeic, Endogeic, Anecic	Briones & Schmidt^28^
Bacteria	Gram-Positive, Gram-Negative, Actinomycetes	Chen et al.^15^
Fungi	Saprophytic, Arbuscular mycorrhizal (AMF)	Chen et al.^15^

Aggregating these species into taxonomic groups permitted more observations and a better understanding of the impact of agricultural practices and possible different effects across taxonomic groups. The taxa were represented by all record points found for their target species used in the search queries, such as bees and wasps in the Arthropod group, or for the taxa when no target species were used, such as nematodes. (Table 1). All biodiversity indicators relating to the species level found in the literature were considered in this review and catalogued for each observation. Indicators found were classified, based on the nature of the indicator, into three main groups for the analysis of biodiversity as either richness, abundance, or compound indicators (e.g., Shannon, Simpson). We then combined these indicators to determine a single biodiversity score. The comparison of taxa with different biodiversity measures might explain some of the variation in the direction of impact, which can be related to the very nature of how these taxa are measured and their specific differences. Aboveground biodiversity had a higher proportion of richness indicators when compared to belowground indicators. The same cannot be observed in belowground taxa, where abundance is the most commonly used biodiversity metric. That has to do with the fact that most studies actually measured the abundance of functional groups within each taxon as a proxy of the biodiversity of the system.

### Literature review

The search was conducted using Boolean operators with different terms in SCOPUS and Web of Science, the two most established search engines in literature reviews, to include the broadest range of agricultural practices possible while restraining the results to the species being targeted per taxonomic group. The search queries were developed to include biodiversity for the taxonomic groups used in this study and can be found in Table 2 and Supplementary Material C. We conducted a pre-review to test the results of our search query in terms of finding the studies of agricultural practices for the taxa chosen in a standardized way. We included results from both croplands and grasslands, with the exception of birds and arthropods for which data found was not sufficient. The search query for belowground biodiversity was different from that for the aboveground taxa and combined both agriculture and grasslands. Search queries for aboveground biodiversity were composed of three main parts: the nature of the practice (e.g., either agriculture or grassland/rangeland and practice); the target species composing the taxa (e.g., bees and wasps for arthropods); and the biodiversity indicator (e.g., richness). The search query for belowground biodiversity also comprised three parts: the agricultural and grazing practices; the taxonomic group; and the biodiversity indicator. Queries were evaluated for both above and belowground compartments because this resulted in more meaningful insights than an analysis based on the cropland and grassland dichotomy for soil. As a result of our preliminary review, we found that combining results for grasslands and agricultural practices for belowground biodiversity and having it separate for aboveground biodiversity yielded more results that were more appropriate to agroecosystems instead of natural areas, which is the aim of our review. Results for both search engines were catalogued and duplicates were removed. With respect to the scope of our work, we only selected studies that focused on the impact of alternative practices on agroecosystems and did not include papers on the impact of practices on the biodiversity of (semi)natural areas. To better represent ecosystem conditions and variations, our inclusion criteria were composed to only select field studies or studies utilizing data from the field.

**Table 2:** Search queries and their respective species

Group	Taxa	Surrogate species	SCOPUS	Web Of Knowledge (all databases)
Croplands	*Arthropods*	Bees	TITLE-ABS-KEY (agricult* AND practice AND bee AND (richness OR abundance))	You searched for: TOPIC: (agricult* AND practice AND bee AND (richness OR abundance))
		Wasps	TITLE-ABS-KEY (agricult* AND practice AND wasp AND (richness OR abundance))	You searched for: TOPIC: (agricult* AND practice AND wasp AND (richness OR abundance))
	*Birds*	Birds	TITLE-ABS-KEY (agricult* AND practice AND bird AND (richness OR abundance))	You searched for: TOPIC: (agricult* AND practice AND bird AND (richness or abundance))
	*Mammals*	Small Rodents	TITLE-ABS-KEY (agricult* AND practice AND rodent AND (richness OR abundance))	You searched for: TOPIC: (agricult* AND practice AND rodent AND (richness OR abundance))
		Bats	TITLE-ABS-KEY (agricult* AND practice AND bat AND (richness OR abundance))	You searched for: TOPIC: (agricult* AND practice AND bat AND (richness OR abundance))
Grasslands	*Mammals*	Small Rodents	TITLE-ABS-KEY (((rangeland AND grassland) OR grassland OR rangeland) AND practice AND rodent AND (richness OR abundance))	You searched for: TOPIC: (((rangeland AND grassland) OR grassland OR rangeland) AND practice AND rodent AND (richness OR abundance))
		Bats	TITLE-ABS-KEY (((rangeland AND grassland) OR grassland OR rangeland) AND practice AND bat AND (richness OR abundance))	You searched for: TOPIC: (((rangeland AND grassland) OR grassland OR rangeland) AND practice AND bat AND (richness OR abundance))
Soil	*Nematodes*	Nematodes	TITLE-ABS-KEY (((agric* AND graz*) OR agric* OR graz*) AND practice AND soil AND nematod* AND (richness OR abundance))	You searched for: TOPIC: (((agric* AND graz*) OR agric* OR graz*) AND practice AND soil AND nematod* AND (richness OR abundance))
	*Earthworms*	Earthworms	TITLE-ABS-KEY (((agric* AND graz*) OR agric* OR graz*) AND practice AND soil AND earthworm AND (richness OR abundance))	You searched for: TOPIC: (((agric* AND graz*) OR agric* OR graz*) AND practice AND soil AND earthworm AND (richness OR abundance))
	*Fungal/Bacterial Biomass Ratio*	Fungal/Bacterial Biomass Ratio	TITLE-ABS-KEY (((agric* AND graz*) OR agric* OR graz*) AND practice AND soil AND bacteri* AND fung* AND ratio AND biomass)	You searched for: TOPIC: (((agric* AND graz*) OR agric* OR graz*) AND practice AND soil AND bacteri* AND fung* AND ratio AND biomass)

**Fig. 4:**
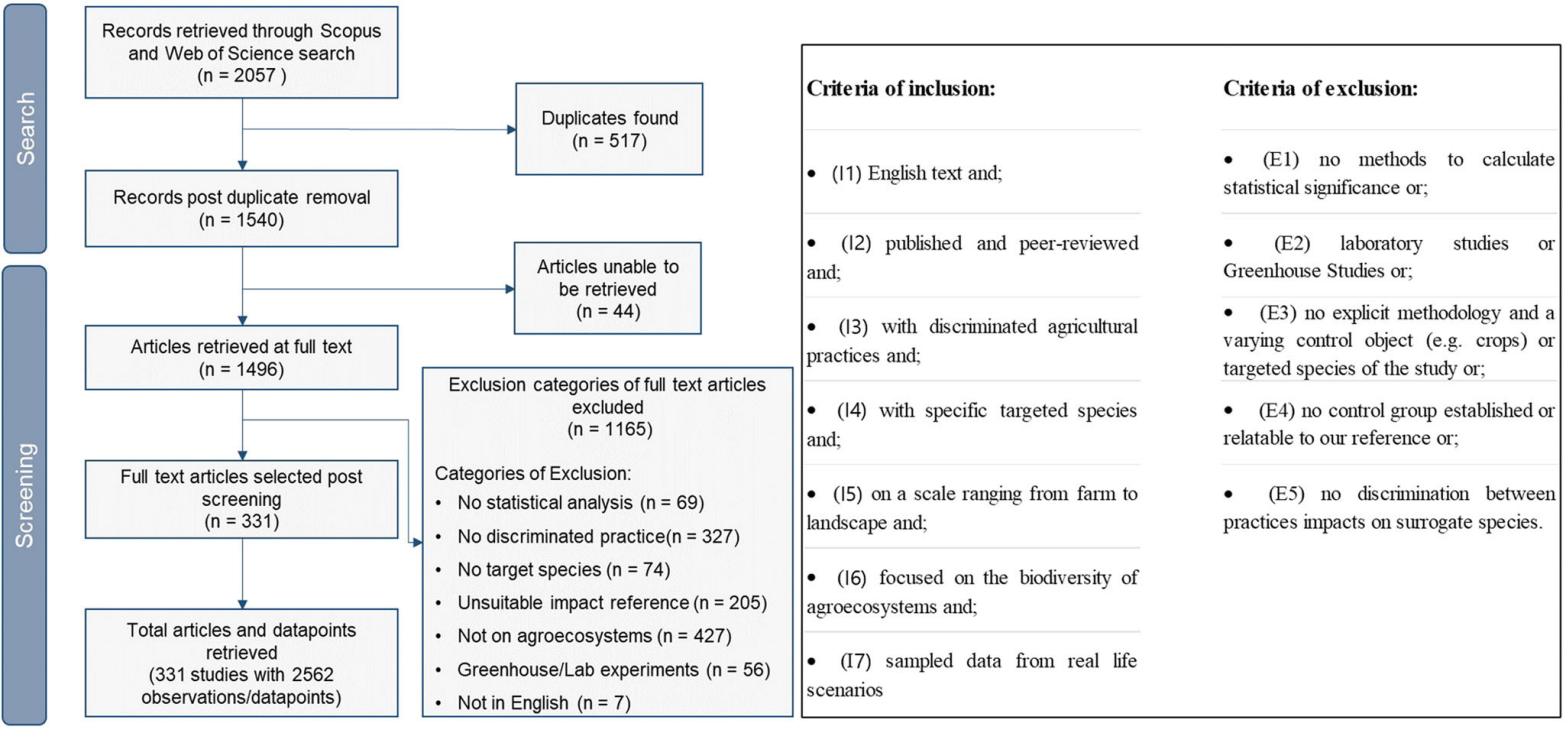
Overview of the criteria and literature screening of the review. Illustration of the research methodology to analyse the impact of different agricultural practices on biodiversity and the criteria of inclusion and exclusion—‘*n*’ represents the number of studies in each step.

The following selection criteria were defined for including a study in the literature review: it needed to be peer-reviewed and published in English; have statistical analysis with documented methodology; and display the impact of alternative practices in such a way that we could discriminate the results for each of the taxa affected. Studies without control groups or with control groups where it was impossible to relate the impact to our intensive practices control setting were excluded (e.g., studies comparing minimum tillage with conservation tillage). Laboratory or greenhouse studies were also excluded. The full list with both inclusion and exclusion criteria is shown in Fig. 4. After an initial review by the first reviewer, the selected papers were then reviewed by a second reviewer among the authors to guarantee all criteria were fulfilled. All included and excluded papers can be found in Supplementary Material D. The impact of the practices was evaluated as positive and negative when the study reported that a given practice significantly influenced a particular taxa (positive as enhancing and negative as detrimental) when compared to intensive practices (Supplementary Material A), and as neutral if results found no statistical significance. Neutral results indicate that impact has not been observed or determined, but it is not an ultimate determination that impact does not exist^44^. The data was then entered into an Excel file containing all untransformed data, referred to as the “impact matrix”, all practices retrieved and their impacts on the targeted species (Supplementary Material E).

### Data transformation and biome assignment

All data regarding the practices found in the literature and their impact on the specific species registered in the “impact matrix” was extracted into an R environment. The data was then filtered in a way that data records were organized so that each practice had a single impact on a single species per configuration of the practice (whether it was applied individually or simultaneously with other practices). These extracted data records were defined by the individual and unique combinations of settings (synchronicity of practices, location), the species and/or taxonomic group affected, and the biodiversity indicator used. Firstly, data transformation permitted combining the results from groups of target species in the same taxa, e.g., bees and wasps data records for arthropods. Data clustered per taxa level had the biodiversity indicators combined (i.e. abundance, richness, or compound) and were classified as positive, negative or neutral. The effects of practices on specific indicators for each of the taxa can be found in Supplementary Material B. Lastly, data records were assigned a biome based on their location. When the administrative units of their location described in the study were written using the GAUL nomenclature, this assignment was done in R (v. 4.0.3) via coding by assigning a biome (adapted from Olson et al. ^56^) based on the location of studies in the Global Administrative Unit Layers geographic base (GAUL, Global administrative boundaries^57^). If the locations were named using nomenclature outside the GAUL list standard, the assigning of biomes to data records was done manually by the reviewers using adapted Olson et al. ^56^ biomes. Each data record relates the impact of a specific observation of a practice on a specific indicator of a specific targeted species/taxonomic group under a specific setting for that biome. R was used for data handling and analysis, using the previously transformed, uniform, and comparable data as well as fitting a structured format for the analysis. All data transformation and clustering can also be observed in the R script.

### Analysis of effects and across biomes

The data records were used to create a distribution of effects for each of the practices on the respective studied taxa. Thus a structured visualization of the proportion of effects was used to assess the practices. Practices were considered majorly positive when the highest proportion of effects, both relative and absolute, was of positive effects and negative when the reverse was observed. Given neutral data records depict no observed effect, but not the absolute lack of effect, we considered shifts from majorly positive/negative to majorly neutral not to be inconsistent, as long as the second highest effect was not of the opposite direction than in other biomes. Practices that had mostly positive/negative effects and a number of data records equal to or higher than 25 were designated to be consistently positive or consistently negative depending on the direction of effects and to enhance biodiversity when positive. To assess whether effects were different in distinct biomes we utilized the distribution of effects for entire groups of practices, e.g. fertilization. The inconsistency of the distribution of effects found in the literature in these cases was then attributed to the group of practices potentially having different effects in different biomes. Groups of practices were considered to not be consistent across biomes when distributions changed from majorly positive to majorly negative, or vice-versa, across biomes.

## Supplementary information

Supplementary Material A

Supplementary Material B

Supplementary Material C

Supplementary Materials D–G

## Data Availability

All data is available in the supplementary materials. Studies used and results collected can be found in Supplementary Materials D and E.

## Code availability

Custom R scripts developed for the analyses and visualizations in this manuscript are available from the corresponding author on request.
