# Supplementary material for: Farming practices to enhance biodiversity across biomes: a systematic review
Source: NPJ Biodivers. 2024 Jan 9;3:1. doi: 10.1038/s44185-023-00034-2 (PMC11332212; doi:10.1038/s44185-023-00034-2)
Supplement: Supplementary file 1 — Supplementary Material A [file 44185_2023_34_MOESM1_ESM.pdf]

Type of Practice	Alternatives to Intensive Farming Practices	Definition of alternative practice in the review	Adapted from	Compared to - Intensive Farming Practice	Definition of intensive practice in the review	Adapted from
Fertilization	No Fertilizer	Agricultural area without application of any type of fertilizer.	Li, S., Li, J., Zhang, B., Li, D., Li, G., Li, Y., 2017. Effect of different organic fertilizers application on growth and environmental risk of nitrate under a vegetable field. Sci Rep 7, 17020. https://doi.org/10.1038/s41598-017-17219-y ; Singh, R., 2012. ; Roy, R. N., & Food and Agriculture Organization of the United Nations (Eds.). (2006). Plant nutrition for food security: A guide for integrated nutrient management. Food and Agriculture Organization of the United Nations.; Gram, G., Roobroeck, D., Pypers, P., Six, J., Merckx, R., & Vanlauwe, B. (2020). Combining organic and mineral fertilizers as a climate-smart integrated soil fertility management practice in sub-Saharan Africa: A meta-analysis. PLoS ONE, 15(9), e0239552. https://doi.org/10.1371/journal.pone.0239552	Synthetic Fertilizer	Application of synthetic fertilizers (inorganic) as input for all the Nitrogen, Potassium and Phosporus needs of the soil and crop. Inorganic fertilizers are referred to as Synthetic Fertilizers.+E2:G6H2F2:G6D2:G6E2:G6	Kumar, R., Kumar, R., & Prakash, O. (2019). The Impact of Chemical Fertilizers on our Environment and Ecosystem (pp. 69–86).<br>Mozumder, P., & Berrens, R. P. (2007). Inorganic fertilizer use and biodiversity risk: An empirical investigation. Ecological Economics, 62(3–4), 538–543. https://doi.org/10.1016/j.ecolecon.2006.07.016<br>Zhang, X., Fang, Q., Zhang, T., Ma, W., Velthof, G. L., Hou, Y., Oenema, O., & Zhang, F. (2020). Benefits and trade-offs of replacing synthetic fertilizers by animal manures in crop production in China: A meta-analysis. Global Change Biology, 26(2), 888–900. https://doi.org/10.1111/gcb.14826. Singh, R., 2012. ; Roy, R. N., & Food and Agriculture Organization of the United Nations (Eds.). (2006).
	Bulky organic fertilizers	Compost and Manure are widespread oragnic fertilizers. They contain some nutrients, but due to their great volume, they also significantly contribute to structuring organic matter addition. This practice includes organic matter incorporation such as straw or plant residues, when applied simultaneously with OM mulch.				
	Concentrated organic fertilizers	These organic fertilizers, which can be in liquid (slurry) or more solid form (commercial organic fertilizers like pellets, cakes etc) have as main function the addition and release of nutreints, less of OM.				
	Inorganic + Bulky Organic Fert	Any type of organic fertilizer combined with the application of synthetic, mineral nitrogen sources usually applied in conventional agriculture. The main N source remains the mineral fertilizer.				
	Bulky Organic + Inorganic Fert	Any type of organic fertilizer combined with the application of synthetic, mineral nitrogen sources usually applied in conventional agriculture. The main N source is the organic fertilizer.				
Crop Diversity	Diverse in Geography	Compilation of diverse farming areas settings such as strip cropping, multicrop field or pixel cropping that involve increasing the amount of crops in the farming area. For the sake of this review, any study that classifies an area as diverse and has more than one crop per field is considered a Diverse Polyculture.	Le Féon, V., Schermann-Legionnet, A., Delettre, Y., Aviron, S., Billeter, R., Bugter, R., Hendrickx, F., & Burel, F. (2010). Intensification of agriculture, landscape composition and wild bee communities: A large scale study in four European countries. *Agriculture, Ecosystems & Environment* , 137(1–2), 143–150. https://doi.org/10.1016/j.agee.2010.01.015 ; Zhong, S., Zeng, H., & Jin, Z. (2015). Responses of Soil Nematode Abundance and Diversity to Long-Term Crop Rotations in Tropical China. Pedosphere, 25(6), 844–852. https://doi.org/10.1016/S1002-0160(15)30065-5	Spatial and Temporal monoculture	Presence of a single crop both in the area where the crop is located and also in time, with no crop rotations happening.	Andow, D. (1983). The extent of monoculture and its effects on insect pest populations with particular reference to wheat and cotton. Agriculture, Ecosystems & Environment, 9(1), 25–35. https://doi.org/10.1016/0167-8809(83)90003-8<br>Bourke, P. M., Evers, J. B., Bijma, P., van Apeldoorn, D. F., Smulders, M. J. M., Kuyper, T. W., Mommer, L., & Bonnema, G. (2021). Breeding Beyond Monoculture: Putting the “Intercrop” Into Crops. Frontiers in Plant Science, 12, 734167. https://doi.org/10.3389/fpls.2021.734167<br>Hennessy, D. A. (2006). On Monoculture and the Structure of Crop Rotations. American Journal of Agricultural Economics, 88(4), 900–914. https://doi.org/10.1111/j.1467-8276.2006.00905.x<br>Plourde, J. D., Pijanowski, B. C., & Pekin, B. K. (2013). Evidence for increased monoculture cropping in the Central United States. Agriculture, Ecosystems & Environment, 165, 50–59. https://doi.org/10.1016/j.agee.2012.11.011
	Diverse Temporal	Specific crop rotations can affect soil biodiversity. For the present review a positive impact of this practice refers to a significantly positive impact of a more diverse rotation compared to the control.				
Miscellaneous	Burning	Intentional and controlled burning of fields during spring (depending on hemisphere).	Little, I. T., Hockey, P. A., & Jansen, R. (2013). A burning issue: Fire overrides grazing as a disturbance driver for South African grassland bird and arthropod assemblage structure and diversity. Biological Conservation, 158, 258–270. https://doi.org/10.1016/j.biocon.2012.09.017; Benson, T. J., Dinsmore, J. J., & Hohman, W. L. (2011). Short-term Effects of Burning and Disking on Songbird Use of Floodplain Conservation Easements. The American Midland Naturalist, 165(2), 257–273. https://doi.org/10.1674/0003-0031-165.2.257; Pernollet, C. A., Guelmami, A., Green, A. J., Curcó Masip, A., Dies, B., Bogliani, G., Tesio, F., Brogi, A., Gauthier-Clerc, M., & Guillemain, M. (2015). A comparison of wintering duck numbers among European rice production areas with contrasting flooding regimes. Biological Conservation, 186, 214–224. https://doi.org/10.1016/j.biocon.2015.03.019; Wagner, S., Fischer, H., Huth, F., 2011. Canopy effects on vegetation caused by harvesting and regeneration treatments. Eur J Forest Res 130, 17–40. https://doi.org/10.1007/s10342-010-0378-z	No practice	Absence of the practices present in this group.	<SAME LITERATURE AS FOR THE PRACTICES THEMSELVES, GIVEN IT IS THEIR ABSENCE THE COUNTERPART> Little, I. T., Hockey, P. A., & Jansen, R. (2013). A burning issue: Fire overrides grazing as a disturbance driver for South African grassland bird and arthropod assemblage structure and diversity. Biological Conservation, 158, 258–270. https://doi.org/10.1016/j.biocon.2012.09.017; Benson, T. J., Dinsmore, J. J., & Hohman, W. L. (2011). Short-term Effects of Burning and Disking on Songbird Use of Floodplain Conservation Easements. The American Midland Naturalist, 165(2), 257–273. https://doi.org/10.1674/0003-0031-165.2.257; Pernollet, C. A., Guelmami, A., Green, A. J., Curcó Masip, A., Dies, B., Bogliani, G., Tesio, F., Brogi, A., Gauthier-Clerc, M., & Guillemain, M. (2015). A comparison of wintering duck numbers among European rice production areas with contrasting flooding regimes. Biological Conservation, 186, 214–224. https://doi.org/10.1016/j.biocon.2015.03.019; Wagner, S., Fischer, H., Huth, F., 2011. Canopy effects on vegetation caused by harvesting and regeneration treatments. Eur J Forest Res 130, 17–40. https://doi.org/10.1007/s10342-010-0378-z
	Flooding	Fields are flooded with water for at least 3.5-4 months.				
	No Canopy Cutting	No canopy trimming of vegetation both agricultural or within agrulcultural production areas. Trees and vegetation are left to grow on their own device.				
Planned Biodiversity Interferences	Natural Buffer Areas	Compilation of implementation of Field margins or Semi or natural areas within the farming area or shared as a minority of the landscape. For farming level studies it represents areas external to the farming area that is maintaned for that purpose (e.g. field margins, natural reserves). For landscape studies it refers to the presence of these areas in the landscape (e.g. adjacent natural or semi natural areas).	Carvell, C., Meek, W.R., Pywell, R.F., Nowakowski, M., 2004. The response of foraging bumblebees to successional change in newly created arable field margins. Biological Conservation 118, 327–339. https://doi.org/10.1016/j.biocon.2003.09.012; Cole, L.J., Brocklehurst, S., Robertson, D., Harrison, W., McCracken, D.I., 2017. Exploring the interactions between resource availability and the utilisation of semi-natural habitats by insect pollinators in an intensive agricultural landscape. Agriculture, Ecosystems & Environment 246, 157–167. https://doi.org/10.1016/j.agee.2017.05.007; Öckinger, E., Smith, H.G., 2006. Semi-natural grasslands as population sources for pollinating insects in agricultural landscapes: Population sources for pollinators. Journal of Applied Ecology 44, 50–59. ; Albrecht, M., Kleijn, D., Williams, N.M., Tschumi, M., Blaauw, B.R., Bommarco, R., Campbell, A.J., Dainese, M., Drummond, F.A., Entling, M.H., Ganser, D., Arjen de Groot, G., Goulson, D., Grab, H., Hamilton, H., Herzog, F., Isaacs, R., Jacot, K., Jeanneret, P., Jonsson, M., Knop, E., Kremen, C., Landis, D.A., Loeb, G.M.,	No practice for biodiversity integration	Absence of any practice either within or in the adjance areas to create better conditions for biodiversity through the stimulation of more natural areas.	<SAME LITERATURE AS FOR THE PRACTICES THEMSELVES, GIVEN IT IS THEIR ABSENCE THE COUNTERPART> Carvell, C., Meek, W.R., Pywell, R.F., Nowakowski, M., 2004. The response of foraging bumblebees to successional change in newly created arable field margins. Biological Conservation 118, 327–339. https://doi.org/10.1016/j.biocon.2003.09.012; Cole, L.J., Brocklehurst, S., Robertson, D., Harrison, W., McCracken, D.I., 2017. Exploring the interactions between resource availability and the utilisation of semi-natural habitats by insect pollinators in an intensive agricultural landscape. Agriculture, Ecosystems & Environment 246, 157–167. https://doi.org/10.1016/j.agee.2017.05.007; Öckinger, E., Smith, H.G., 2006. Semi-natural grasslands as population sources for pollinating insects in agricultural landscapes: Population sources for pollinators. Journal of Applied Ecology 44, 50–59. ; Albrecht, M., Kleijn, D., Williams, N.M., Tschumi, M., Blaauw, B.R., Bommarco, R., Campbell, A.J., Dainese, M., Drummond, F.A.,
	Unproductive Biodiversity Zones	Set of practices involving implementing vegetation strips, such as flower strips, within the agricultural zone to provide foraging or safe areas for local and/or agriculturally relevant species.				
Pesticide Use	No Insecticide Use	No application of chemicals used to control insect populations, also known as insecticides .	Pimentel, D., McLaughlin, L., Zepp, A., Lakitan, B., Kraus, T., Kleinman, P., Vancini, F., Roach, W.J., Graap, E., Keeton, W.S., Selig, G., 1991. Environmental and Economic Effects of Reducing Pesticide Use. BioScience 41, 402–409. https://doi.org/10.2307/1311747; Zhang, W., 2018. Global pesticide use: Profile, trend, cost / benefit and more 28.	Full use of Pesticides - Herbicides, Fungicides and Insecticides	Application of fungicides, herbicides and insectides in the crop fields. We assume the use of pesticide to be as instructed on label, following dosages as intructed by manufacturer. We also assume application will be done as instructed on label.	De, A., Bose, R., Kumar, A., & Mozumdar, S. (2014). Worldwide Pesticide Use. In A. De, R. Bose, A. Kumar, & S. Mozumdar (Eds.), Targeted Delivery of Pesticides Using Biodegradable Polymeric Nanoparticles (pp. 5–6). Springer India. https://doi.org/10.1007/978-81-322-1689-6_2<br>Zhang, W. (2018). Global pesticide use: Profile, trend, cost / benefit and more.
	No Herbicide Use	No application of chemicals used to control weed population, also known as herbicides .				
	No Fungicide Use	No application of chemicals used to control fungal population, also known as fungicides.				
GMO	No GMO Use	The absence of any crop that has been genetically altered to have perceived benefitial agricultural traits. This category does not include selective breeding sourced crops.	Agostini, M.G., Roesler, I., Bonetto, C., Ronco, A.E., Bilenca, D., 2020. Pesticides in the real world: The consequences of GMO-based intensive agriculture on native amphibians. Biological Conservation 241, 108355. https://doi.org/10.1016/j.biocon.2019.108355; Caswell, J.A., n.d. An evaluation of risk analysis as applied to agricultural biotechnology (with a case study of gmo labeling) 9.	Use of GMOs in the field.	Presence of genetically modified organisms in the field to improve agricultural conditions, such as, but not limited to yield and resistence.	Van Acker, R., Rahman, M. M., & Cici, S. Z. H. (2017). Pros and Cons of GMO Crop Farming. In R. Van Acker, M. M. Rahman, & S. Z. H. Cici, Oxford Research Encyclopedia of Environmental Science. Oxford University Press. https://doi.org/10.1093/acrefore/9780199389414.013.217; Agostini, M.G., Roesler, I., Bonetto, C., Ronco, A.E., Bilenca, D., 2020. Pesticides in the real world: The consequences of GMO-based intensive agriculture on native amphibians. Biological Conservation 241, 108355. https://doi.org/10.1016/j.biocon.2019.108355; Caswell, J.A., n.d. An

Tillage	Minimum Tillage	Use of only minimum secondary tillage and no primary tillage and without turning over the soil.	Derksen, D. A., Lafond, G. P., Thomas, A. G., Loeppky, H. A. and Swanton, C. J. (1993) "Impact of Agronomic Practices on Weed Communities: Tillage Systems," Weed Science. Cambridge University Press, 41(3), pp. 409–417. doi: 10.1017/S0043174500052127. ; Carter, M. R. (2005). CONSERVATION TILLAGE. In D. Hillel (Ed.), Encyclopedia of Soils in the Environment (pp. 306–311). Elsevier. https://doi.org/10.1016/B0-12-348530-4/00270-8; Horwath, W. R., & Kuzyakov, Y. (2018). Chapter Three—The Potential for Soils to Mitigate Climate Change Through Carbon Sequestration. In W. R. Horwath & Y. Kuzyakov (Eds.), Developments in Soil Science (Vol. 35, pp. 61–92). Elsevier. https://doi.org/10.1016/B978-0-444-63865-6.00003-X; Mitchell, J. P., Pettygrove, G. S., Upadhyaya, S., Shrestha, A., Fry, R., Roy, R., Hogan, P., Vargas, R., & Hembree, K. (2009). Classification of Conservation Tillage Practices in California Irrigated Row Crop Systems. University of California, Agriculture and Natural Resources. https://doi.org/10.3733/ucanr.8364 ; McCalla, T.M., Army, T.J., 1961. https://doi.org/10.1016/S0065-2113(08)60958-X	Intensive tillage	Tilling method leaving less than 15% of crop residue cover in the field, involving soil inversion and having both a primary and secondary tillage operations. Conventional tillage is referred to here as intensive tillage.	Miller, P. R., Buschena, D. E., Jones, C. A., & Holmes, J. A. (2008). Transition from Intensive Tillage to No-Tillage and Organic Diversified Annual Cropping Systems. Agronomy Journal, 100(3), 591–599. https://doi.org/10.2134/agronj2007.0190; Eurostat Agri-environmental indicator - tillage practices at https://ec.europa.eu/eurostat/statistics-explained/index.php?title=Glossary:Conventional_tillage
	Zero Tillage	No tilling applied to the agricultural area.				
	Conservation Tillage	While conservation tillage might be considered a generic umbrella including minimun and zero tillage we consider the defintion including residue management criteria. Hence, conservation tillage its the use of reduced tillage and residue management to maintain plant residues on a minimum of 30% of soil surface post tillage.				
	Stubble mulch tillage/stubble mulch farming	Minimum tillage leaving the stubble or crop residue on agricultural soil during the fallow period.				
Soil cover	Synthetic Mulching	A mulch may take many forms: loose particles of organic or inorganic matter spread over the soil or sheets of artificial or natural materials laid on the soil surface. Even spray-on mulches have been developed that form a thin latex-based film on the soil surface; organic mulching uses organic material while synthetic utilize ones such as plastic.	Bond, W., & Grundy, A. C. (2001). *Non-chemical weed management in organic farming systems* . 23. ; Roy, R. N., & Food and Agriculture Organization of the United Nations (Eds.). (2006). Plant nutrition for food security: A guide for integrated nutrient management. Food and Agriculture Organization of the United Nations. ; Campbell, J. W., Grodsky, S. M., Monroe, A. P., & Martin, J. A. (2021). Bee (Apoidea) community response to perennial grass treatments managed for livestock production and conservation. Agriculture, Ecosystems & Environment, 313, 107391. https://doi.org/10.1016/j.agee.2021.107391<br>Du, S., You, S. H., Bao, J., Gegentu, Jia, Y. S., & Cai, Y. M. (2019). Evaluation of the growth performance and meat quality of Mongolian lamb fed grass, hay or pellets of Inner Mongolian native grass. Small Ruminant Research, 181, 34–38. https://doi.org/10.1016/j.smallrumres.2019.10.008 ; McLaughlin, A., Mineau, P., 1995. The impact of agricultural practices on biodiversity. Agriculture, Ecosystems & Environment 55, 201–212. https://doi.org/10.1016/0167-8809(95)00609-V ; Tälle, M., Deák, B., Poschlod, P., Valkó, O., Westerberg, L., Milberg, P., 2016. Grazing vs. mowing: A meta-analysis of biodiversity benefits for grassland management. Agriculture, Ecosystems & Environment 222, 200–212. https://doi.org/10.1016/j.agee.2016.02.008	Absence of any soil cover	Absence of any soil cover either as mulching or as intercropping. Soil is left unprotected and exposed apart from the crop.	<SAME LITERATURE AS FOR THE PRACTICES THEMSELVES, GIVEN IT IS THEIR ABSENCE THE COUNTERPART> Bond, W., & Grundy, A. C. (2001). *Non-chemical weed management in organic farming systems* . 23. ; Roy, R. N., & Food and Agriculture Organization of the United Nations (Eds.). (2006). Plant nutrition for food security: A guide for integrated nutrient management. Food and Agriculture Organization of the United Nations. ; Campbell, J. W., Grodsky, S. M., Monroe, A. P., & Martin, J. A. (2021). Bee (Apoidea) community response to perennial grass treatments managed for livestock production and conservation. Agriculture, Ecosystems & Environment, 313, 107391. https://doi.org/10.1016/j.agee.2021.107391<br>Du, S., You, S. H., Bao, J., Gegentu, Jia, Y. S., & Cai, Y. M. (2019). Evaluation of the growth performance and meat quality of Mongolian lamb fed grass, hay or pellets of Inner Mongolian native grass. Small Ruminant Research, 181, 34–38. https://doi.org/10.1016/j.smallrumres.2019.10.008 ; McLaughlin, A., Mineau, P., 1995. The impact of agricultural practices on biodiversity. Agriculture, Ecosystems & Environment 55, 201–212. https://doi.org/10.1016/0167-8809(95)00609-V ; Tälle, M., Deák, B., Poschlod, P., Valkó, O., Westerberg, L., Milberg, P., 2016. Grazing vs. mowing: A meta-analysis of biodiversity benefits for grassland management. Agriculture, Ecosystems & Environment 222, 200–212. https://doi.org/10.1016/j.agee.2016.02.008
	Organic matter mulching					
	Living mulch (cover crop, green manure)	Usually cover crops, with and without leguminous plants, are grown as living muilch and incorporated into the soil as nutrient and structural contribution.				
	Native Grass/Vegetation	Presence and maintenance of vegetation (flora) that is naturally present or expected in a given environment for grasslands.				
	No Mowing	Absence of mowing as a method to control weeds on both agricultural range- and croplands. This can be the case for uncultivated fieldstrips, interrows in vineyards as well as meadows .				
Irrigation	No Irrigation	Agricultural area without application of any type of irrigation, a part from natural occuring precipitation.	Masri, M.I., Ramadan, B.S.B., El-Shafai, A.M.A., El-Kady, M.S., n.d. Effect of water stress and fertilization on yield and quality of sugar beet under drip and sprinkler irrigation systems in sandy soil 14.; Albaji, M., Golabi, M., Boroomand Nasab, S., Zadeh, F.N., 2015. Investigation of surface, sprinkler and drip irrigation methods based on the parametric evaluation approach in Jaizan Plain. Journal of the Saudi Society of Agricultural Sciences 14, 1–10. https://doi.org/10.1016/j.jssas.2013.11.001 ; Sammis, T.W., 1980. Comparison of Sprinkler, Trickle, Subsurface, and Furrow Irrigation Methods for Row Crops 1. Agron.j. 72, 701–704. https://doi.org/10.2134/agronj1980.00021962007200050002x; Pereira, L.S., Oweis, T., Zairi, A., 2002. Irrigation management under water scarcity. Agricultural Water Management 57, 175–206. https://doi.org/10.1016/S0378-3774(02)00075-6 ; Masri, M.I., Ramadan, B.S.B., El-Shafai, A.M.A., El-Kady, M.S., n.d. Effect of water stress and fertilization on yield and quality of sugar beet under drip and sprinkler irrigation systems in sandy soil 14.; Albaji, M., Golabi, M., Boroomand Nasab, S., Zadeh, F.N., 2015. Investigation of surface, sprinkler and drip irrigation methods based on the parametric evaluation approach in Jaizan Plain. Journal of the Saudi Society of Agricultural Sciences 14, 1–10. https://doi.org/10.1016/j.jssas.2013.11.001	Intensive irrigation	Irrigation (complimentary to rainfed) of arable fields and crops with the use of surface irrigation. Our definition is composed of a irrigation by central pivot, basin irrigation, border strip irrigation or gated pipe supply systems.	Maisiri, N., Senzanje, A., Rockstrom, J., & Twomlow, S. J. (2005). On farm evaluation of the effect of low cost drip irrigation on water and crop productivity compared to conventional surface irrigation system. Physics and Chemistry of the Earth, Parts A/B/C, 30(11–16), 783–791. https://doi.org/10.1016/j.pce.2005.08.021; Walker, W. R., & Skogerboe, G. V. (1987). Surface Irrigation: Theory and Practice.
	Irrigation - Sprinkler	Sprinkler based irrigation.				
	Irrigation - Subsurface	Use of irrigation techniques applied directly under the surface of agricultural ground with drip techniques.				
	Irrigation - Furrow	Furrow irrigation is a method where water is applied to furrows using small discharges to favour water infiltration while advancing down the field.				
	Irrigation - Drip/Trickle	Use of restrained minimum water needed flow for plants and soil. Applied on the surface of the soil.				
Grazing	Rotanional Grazing	Use of multiple paddocks,minimun of three, for alternating grazing and resting periods.	DORROUGH, J., MCINTYRE, S., BROWN, G., STOL+D32:E34, J., BARRETT, G., & BROWN, A. (2012). Differential responses of plants, reptiles and birds to grazing management, fertilizer and tree clearing. Austral Ecology, 37(5), 569–582. https://doi.org/10.1111/j.1442-9993.2011.02317.x; Ranellucci, C. L., Koper, N., & Henderson, D. C. (2012). Twice-Over Rotational Grazing and Its Impacts on Grassland Songbird Abundance and Habitat Structure. Rangeland Ecology & Management, 65(2), 109–118. https://doi.org/10.2111/rem-d-11-00053.1 ; Batáry, P., Báldi, A., & Erdős, S. (2006). Grassland versus non-grassland bird abundance and diversity in managed grasslands: local, landscape and regional scale effects. Biodiversity and Conservation, 16(4), 871–881. https://doi.org/10.1007/s10531-006-9135-5; Loe, L. E., Mysterud, A., Stien, A., Steen, H., Evans, D. M., & Austrheim, G. (2006). Positive short-term effects of sheep grazing on the alpine avifauna. Biology Letters, 3(1), 110–112. https://doi.org/10.1098/rsbl.2006.0571 ;	Intensive grazing	ve grazing is defined in this review as any grazing happening with a stocking rate of >1Livestock U	DORROUGH, J., MCINTYRE, S., BROWN, G., STOL+D32:E34, J., BARRETT, G., & BROWN, A. (2012). Differential responses of plants, reptiles and birds to grazing management, fertilizer and tree clearing. Austral Ecology, 37(5), 569–582. https://doi.org/10.1111/j.1442-9993.2011.02317.x; Ranellucci, C. L., Koper, N., & Henderson, D. C. (2012). Twice-Over Rotational Grazing and Its Impacts on Grassland Songbird Abundance and Habitat Structure. Rangeland Ecology & Management, 65(2), 109–118. https://doi.org/10.2111/rem-d-11-00053.1 ; Batáry, P., Báldi, A., & Erdős, S. (2006). Grassland versus non-grassland bird abundance and diversity in managed grasslands: local, landscape and regional scale effects. Biodiversity and Conservation, 16(4), 871–881. https://doi.org/10.1007/s10531-006-9135-5; Loe, L. E., Mysterud, A., Stien, A., Steen, H., Evans, D. M., & Austrheim, G. (2006). Positive short-term effects of sheep grazing on the alpine avifauna. Biology Letters, 3(1), 110–112. https://doi.org/10.1098/rsbl.2006.0571 ; Nelson, K. S., Gray, E. M., & Evans, J. R. (2011). Finding solutions for bird restoration and
	Extensive Grazing	Lower number of livestock used. The definition of "lower" varies but stays mostly somewhere in between 0.5Livestock Unit/ha vs. intensive being >1Livestock Unit/ha.				

	No Grazing	No grazing by any kind of livestock.	Nelson, K. S., Gray, E. M., & Evans, J. R. (2011). Finding solutions for bird restoration and livestock management: comparing grazing exclusion levels. Ecological Applications, 21(2), 547–554. https://doi.org/10.1890/10-0032.1; Loe, L. E., Mysterud, A., Stien, A., Steen, H., Evans, D. M., & Austrheim, G. (2006). Positive short-term effects of sheep grazing on the alpine avifauna. Biology Letters, 3(1), 110–112. https://doi.org/10.1098/rsbl.2006.0571			Nelson, K. S., Gray, E. M., & Evans, J. R. (2011). Finding solutions for bird restoration and livestock management: comparing grazing exclusion levels. Ecological Applications, 21(2), 547–554. https://doi.org/10.1890/10-0032.1; Loe, L. E., Mysterud, A., Stien, A., Steen, H., Evans, D. M., & Austrheim, G. (2006). Positive short-term effects of sheep grazing on the alpine avifauna. Biology Letters, 3(1), 110–112. https://doi.org/10.1098/rsbl.2006.0571; Whitt, C. (n.d.). Rotational Grazing Adoption by Cow-Calf Operations.
Livestock Care	Vaccines - Livestock	Regular, (when required) repeated treatment with different types of vaccines as precautionary protection against diseases and economic loss in lifestock .	Doeschl-Wilson, A., Knap, P. W., Opriessnig, T., & More, S. J. (2021). Review: Livestock disease resilience: From individual to herd level. Sustainable Livestock Systems for High-Producing Animals, 15, 100286. https://doi.org/10.1016/j.animal.2021.100286 ; Moreno, L., n.d. Chapter 24 - Specific Veterinary Drug Residues of Concern in Meat Production 23.	No treatment	No vaccines or health treatment applied to livestock	<SAME LITERATURE AS FOR THE PRACTICES THEMSELVES, GIVEN IT IS THEIR ABSENCE THE COUNTERPART> Doeschl-Wilson, A., Knap, P. W., Opriessnig, T., & More, S. J. (2021). Review: Livestock disease resilience: From individual to herd level. Sustainable Livestock Systems for High-Producing Animals, 15, 100286. https://doi.org/10.1016/j.animal.2021.100286 ; Moreno, L., n.d. Chapter 24 - Specific Veterinary Drug Residues of Concern in Meat Production 23.
	Vermicides - Livestock	Frequent treatment of the animal with the pharmacologic product. Application of anthelmintics, usually once or twice per year, is traditional in the farming culture and they are one of the most commonly administered veterinary livestock drugs in most countries.				
