# Supplementary material for: Farming practices to enhance biodiversity across biomes: a systematic review
Source: NPJ Biodivers. 2024 Jan 9;3:1. doi: 10.1038/s44185-023-00034-2 (PMC11332212; doi:10.1038/s44185-023-00034-2)

Supplementary material B

List of files and description

- ***I-) List of species, indicators and their respective datapoints:*** List of all data records per taxa, species, indicator and practice without distinction of impact

- ***II-) relative distr:*** Visualization of the impact of each practice on each taxa based on the indicator affected as a relative distribution of total data records retrieved for that practice

- ***III-) Absolute distr:*** Visualization of the impact of each practice on each taxa based on the indicator affected as the absolute number of data records retrieved for that practice

- ***IV-) impact per ind:*** Mirrored, more detailed version of figure 2, displaying the parcel of each indicator in the total effect size of a practice on a taxa, as positive, negative or neutral

TERMINOLOGY IN DATABASE – USED IN THE MANUSCRIPT

In database	Used in Manuscript
Crop Diversity - Diverse in Geography	Spatial
Crop Diversity - Diverse Temporal	Temporal
Bulky Organic + Inorganic Fert	Organic Supplemented
Bulky Organic Fertilizers	Organic
Concentrated Organic Fertilizers	Concentrated Organic
Inorganic + Bulky Organic Fert	Synt. Supplemented
No Fertilizer	No Fert.
No GMO Use	No GMO
Extensive Grazing	Extensive
No Grazing	No Grazing
Rotational Grazing	Rotational
Irrigation - Furrow	Furrow
Irrigation - Sprinkler	Sprinkler
No Irrigation	Rainfed
No Fungicide Use	No Fungicide
No Herbicide Use	No Herbicide
No Insecticide Use	No Insecticide
Natural Buffer Areas	Buffer Areas
Unproductive Biodiversity Zones	Enhacement Strips
Cover Crop / Green Manure / Living Mulch	Cover Crop
Native Grass/Vegetation	Native Vegetation
No Mowing	No Mowing
Organic Matter Mulching	Organic Matter Mulching
Synthetic Mulching	Synthetic Mulching
Conservation Tillage	Conservation
Minimum Tillage	Minimum
Stubble Mulch Tillage/Stubble Mulch Farming	Stubble Mulch
Zero Tillage	Zero
Burning	Burning

Flooding	Flooding
No Canopy Cutting	Unmanaged Canopy

I-) List of species, indicators and their respective datapoints

Taxa	Target Species	Biodiversity Indicator	Biodiversity Indicator Category	NumberData points
Arthropods	Bees	Abundance	Abundance	24
Arthropods	Bees	Evenness	Compound	4
Arthropods	Bees	Richness	Richness	51
Arthropods	Wasps	Abundance	Abundance	12
Arthropods	Wasps	Richness	Richness	10
Arthropods	Wasps	Shannon Index	Compound	2
Bacteria	Actinomycetes	Abundance	Abundance	10
Bacteria	Actinomycetes	EL-FAME abundnace	Abundance	12
Bacteria	Actinomycetes	PLFA Abundance	Abundance	12
Bacteria	Actinomycetes	PLFA Relative Abundance	Abundance	6
Bacteria	G-	EL-FAME abundnace	Abundance	12
Bacteria	G-	PLFA Abundance	Abundance	14
Bacteria	G-	PLFA Relative Abundance	Abundance	7
Bacteria	G+	EL-FAME abundnace	Abundance	12
Bacteria	G+	PLFA Abundance	Abundance	14
Bacteria	G+	PLFA Relative Abundance	Abundance	7
Bacteria	General	Abundance	Abundance	39
Bacteria	General	Amino Acid Abundance	Abundance	9
Bacteria	General	Biomass	Abundance	19
Bacteria	General	Biomass Carbon	Abundance	8
Bacteria	General	Chao1	Compound	1
Bacteria	General	DNA Abundance	Abundance	2
Bacteria	General	EL-FAME Abundance	Abundance	6
Bacteria	General	EL-FAME abundnace	Abundance	12

Bacteria	***General***	***Fisher's Alpha Diversity***	***Compound***	***1***
Bacteria	***General***	***mol PLFA-C%***	***Abundance***	***1***
Bacteria	***General***	***PLFA Abundance***	***Abundance***	***44***
Bacteria	***General***	***PLFA Relative abundace***	***Abundance***	***1***
Bacteria	***General***	***PLFA Relative Abundance***	***Abundance***	***6***
Bacteria	***General***	***Relative PLFA Abundance***	***Abundance***	***3***
Bacteria	***General***	***richness***	***Richness***	***1***
Bacteria	***General***	***rRNA Abundance***	***Abundance***	***9***
Bacteria	***General***	***Shannon***	***Compound***	***7***
Bacteria	***General***	***Shannon Diversity***	***Compound***	***9***
Bacteria	***General***	***Shannon functional Diversity***	***Compound***	***1***
Bacteria	***General***	***SIR Biomass***	***Abundance***	***2***
Bacteria	***General***	***Taxa richness***	***Richness***	***4***
Bacteria	***General***	***Taxa Richness***	***Richness***	***3***
Birds	***A. arvensis***	***Abundance***	***Abundance***	***3***
Birds	***Alauda arvensis***	***Abundance***	***Abundance***	***1***
Birds	***American pipit***	***Abundance***	***Abundance***	***2***
Birds	***Black phoebe***	***Abundance***	***Abundance***	***1***
Birds	***Blue-headed wagtails***	***Abundance***	***Abundance***	***3***
Birds	***Buteo buteo***	***Abundance***	***Abundance***	***3***
Birds	***Coopers hawk***	***Abundance***	***Abundance***	***1***
Birds	***Cranes***	***Density***	***Abundance***	***3***
Birds	***Dickcissel***	***Abundance***	***Abundance***	***2***
Birds	***Eastern meadowlark***	***Abundance***	***Abundance***	***4***
Birds	***Emberiza calandra***	***Abundance***	***Abundance***	***1***
Birds	***Feckle-breasted thornbird***	***Abundance***	***Abundance***	***2***
Birds	***Geese***	***Density***	***Abundance***	***3***

Birds	***Granivores***	***Abundance***	***Abundance***	***10***
Birds	***Gras wren***	***Abundance***	***Abundance***	***2***
Birds	***Grasshopper sparrow***	***Abundance***	***Abundance***	***1***
Birds	***grassland birds***	***Abundance***	***Abundance***	***4***
Birds	***grassland birds***	***Richness***	***Richness***	***4***
Birds	***Grassland yellow-finch***	***Abundance***	***Abundance***	***2***
Birds	***Great pampa-finch***	***Abundance***	***Abundance***	***2***
Birds	***Henslow's sparrow***	***Abundance***	***Abundance***	***1***
Birds	***Horned lark***	***Abundance***	***Abundance***	***5***
Birds	***House finch***	***Abundance***	***Abundance***	***1***
Birds	***Insectivores***	***Abundance***	***Abundance***	***10***
Birds	***Killdeer***	***Abundance***	***Abundance***	***1***
Birds	***Linaria cannabina***	***Abundance***	***Abundance***	***1***
Birds	***Long-legged wader***	***Density***	***Abundance***	***3***
Birds	***Longspurs***	***Abundance***	***Abundance***	***3***
Birds	***Marsh wren***	***Abundance***	***Abundance***	***1***
Birds	***Motacilla flava***	***Abundance***	***Abundance***	***1***
Birds	***Mourning dove***	***Abundance***	***Abundance***	***1***
Birds	***non-grassland birds***	***Abundance***	***Abundance***	***4***
Birds	***non-grassland birds***	***Richness***	***Richness***	***4***
Birds	***Nothern harrier***	***Abundance***	***Abundance***	***1***
Birds	***Re-tailed hawk***	***Abundance***	***Abundance***	***1***
Birds	***Red-capped wren-spinetail***	***Abundance***	***Abundance***	***4***
Birds	***Red-winged blackbird***	***Abundance***	***Abundance***	***2***
Birds	***Savannah sparrow***	***Abundance***	***Abundance***	***2***
Birds	***Shorebirds***	***Density***	***Abundance***	***3***
Birds	***Skylark***	***Abundance***	***Abundance***	***3***
Birds	***Song sparrow***	***Abundance***	***Abundance***	***1***
Birds	***specialist species***	***Abundance***	***Abundance***	***5***
Birds	***Sturnus vulgaris***	***Abundance***	***Abundance***	***3***

Birds	***Sylvia communis***	***Abundance***	***Abundance***	***1***
Birds	***tbd***	***Abundance***	***Abundance***	***60***
Birds	***tbd***	***Alpha diversity***	***Richness***	***6***
Birds	***tbd***	***Density***	***Abundance***	***4***
Birds	***tbd***	***Richness***	***Richness***	***45***
Birds	***tbd***	***Shannon-Weiner Index***	***Compound indicator***	***9***
Birds	***tbd***	***Simpson Index***	***Compound indicator***	***2***
Birds	***tbd***	***Sorensons Quantitative Index***	***Compound indicator***	***1***
Birds	***Turdus merula***	***Abundance***	***Abundance***	***1***
Birds	***Turkey vulture***	***Abundance***	***Abundance***	***1***
Birds	***Vesper sparrow***	***Abundance***	***Abundance***	***1***
Birds	***Waterbirds***	***Richness***	***Richness***	***3***
Birds	***Western meadowlark***	***Abundance***	***Abundance***	***1***
Birds	***White-tailed kite***	***Abundance***	***Abundance***	***1***
Birds	***Yellow-headed blackbird***	***Abundance***	***Abundance***	***1***
Birds	***Yellow-winged blackbird***	***Abundance***	***Abundance***	***2***
Earthworms	***Anecic***	***Abundance***	***Abundance***	***45***
Earthworms	***Anecic***	***Abundance***		
Earthworms	***Anecic***	***Relative Abundance***	***Abundance***	***3***
Earthworms	***Anecic***	***Shannon***	***Compound***	***7***
Earthworms	***Endogeic***	***Abundance***	***Abundance***	***47***
Earthworms	***Endogeic***	***Abundance***		
Earthworms	***Endogeic***	***PLFA Abundance***	***Abundance***	***2***
Earthworms	***Endogeic***	***Relative Abundance***	***Abundance***	***3***
Earthworms	***Endogeic***	***Shannon***	***Compound***	***7***
Earthworms	***Epi-Endogeic***	***Abundance***	***Abundance***	***15***
Earthworms	***Epigeic***	***Abundance***	***Abundance***	***34***
Earthworms	***Epigeic***	***Abundance***		

Earthworms	***Epigeic***	***PLFA Abundance***	***Abundance***	***2***
Earthworms	***Epigeic***	***Relative Abundance***	***Abundance***	***3***
Earthworms	***Epigeic***	***Shannon***	***Compound***	***7***
Earthworms	***General***	***Abundance***	***Abundance***	***150***
Earthworms	***General***	***Abundance***		
Earthworms	***General***	***PLFA Abundance***	***Abundance***	***2***
Earthworms	***General***	***richness***	***Richness***	***3***
Earthworms	***General***	***Shannon***	***Compound***	***35***
Earthworms	***General***	***Species Diversity***	***Richness***	***7***
Earthworms	***General***	***Species Richness***	***Richness***	***34***
F:B Ratio	***General***	***Abundance***	***Abundance***	***11***
F:B Ratio	***General***	***Abundance Ratio***	***Ratio***	***13***
F:B Ratio	***General***	***Abundance***		
F:B Ratio	***General***	***Amino Acid Abundance Ratio***	***Ratio***	***6***
F:B Ratio	***General***	***Biomass Carbon***	***Abundance***	***4***
F:B Ratio	***General***	***DNA Abundance Ratio***	***Ratio***	***2***
F:B Ratio	***General***	***EL-FAME Abundance***	***Abundance***	***4***
F:B Ratio	***General***	***EL-FAME Abundance Ratio***	***Ratio***	***3***
F:B Ratio	***General***	***EL-FAME abundnace Ratio***	***Ratio***	***12***
F:B Ratio	***General***	***mol PLFA-C%***	***Abundance***	***1***
F:B Ratio	***General***	***PLFA Abundance***	***Abundance***	***16***
F:B Ratio	***General***	***PLFA Abundance Ratio***	***Ratio***	***38***
F:B Ratio	***General***	***PLFA Ratio***	***Ratio***	***4***
F:B Ratio	***General***	***PLFA Relative abundace***	***Abundance***	***1***
F:B Ratio	***General***	***Ratio***	***Ratio***	***6***
F:B Ratio	***General***	***Relative PLFA Abundance Ratio***	***Abundance***	***3***

F:B Ratio	***General***	***rRNA Abundance***	***Abundance***	***3***
F:B Ratio	***General***	***rRNA Abundance Ratio***	***Ratio***	***7***
Fungi	***AMF***	***EL-FAME Abundance***	***Abundance***	***2***
Fungi	***AMF***	***EL-FAME abundnace***	***Abundance***	***12***
Fungi	***AMF***	***PLFA Abundance***	***Abundance***	***10***
Fungi	***AMF***	***PLFA Relative abundace***	***Abundance***	***1***
Fungi	***AMF***	***PLFA Relative Abundance***	***Abundance***	***1***
Fungi	***General***	***Abundance***	***Abundance***	***38***
Fungi	***General***	***Amino Acid Abundance***	***Abundance***	***11***
Fungi	***General***	***Biomass***	***Abundance***	***22***
Fungi	***General***	***Biomass Carbon***	***Abundance***	***9***
Fungi	***General***	***Chao1***	***Compound***	***1***
Fungi	***General***	***DNA Abundance***	***Abundance***	***2***
Fungi	***General***	***EL-FAME Abundance***	***Abundance***	***6***
Fungi	***General***	***EL-FAME abundnace***	***Abundance***	***12***
Fungi	***General***	***Fisher's Alpha Diversity***	***Compound***	***1***
Fungi	***General***	***hyphal length***	***Abundance***	***1***
Fungi	***General***	***Inverse Simpson's index***	***Compound***	***4***
Fungi	***General***	***mol PLFA-C%***	***Abundance***	***1***
Fungi	***General***	***PLFA Abundance***	***Abundance***	***51***
Fungi	***General***	***PLFA Relative abundace***	***Abundance***	***1***
Fungi	***General***	***PLFA Relative Abundance***	***Abundance***	***7***
Fungi	***General***	***Relative PLFA Abundance***	***Abundance***	***3***
Fungi	***General***	***richness***	***Richness***	***5***
Fungi	***General***	***rRNA Abundance***	***Abundance***	***9***

Fungi	***General***	***Shannon Diversity***	***Compound***	***9***
Fungi	***General***	***SIR Biomass***	***Abundance***	***2***
Fungi	***Saprophytic***	***EL-FAME abundnace***	***Abundance***	***12***
Fungi	***Saprophytic***	***PLFA Abundance***	***Abundance***	***4***
Fungi	***Saprophytic***	***PLFA Relative abundace***	***Abundance***	***1***
Mammals	***Bat***	***Bat activity***	***Abundance***	***4***
Mammals	***Bat***	***Bat passes***	***Abundance***	***4***
Mammals	***Bat***	***Shannon Index***	***Compound***	***1***
Mammals	***Bat***	***Species Abundance***	***Abundance***	***24***
Mammals	***Bat***	***Species Richness***	***Richness***	***23***
Mammals	***Beaver***	***Species Abundance***	***Abundance***	***1***
Mammals	***coypus***	***Species Abundance***	***Abundance***	***2***
Mammals	***Gerbil***	***Shannon***	***Compound***	***1***
Mammals	***Gerbil***	***Species Abundance***	***Abundance***	***1***
Mammals	***Gerbil***	***Species Richness***	***Richness***	***1***
Mammals	***Gopher***	***Species Abundance***	***Abundance***	***2***
Mammals	***Hamster***	***Species Abundance***	***Abundance***	***1***
Mammals	***Mice***	***Capture Proportion***	***Abundance***	***1***
Mammals	***Mice***	***Shannon***	***Compound***	***5***
Mammals	***Mice***	***Shannon and Simpson Index***	***Compound***	***2***
Mammals	***Mice***	***Shannon Index***	***Compound***	***1***
Mammals	***Mice***	***Species Abundance***	***Abundance***	***29***
Mammals	***Mice***	***Species Abundance***	***NA***	***1***
Mammals	***Mice***	***Species Richness***	***Richness***	***11***
Mammals	***Mice***	***Species Richness***	***NA***	***1***
Mammals	***Opossum***	***Species Abundance***	***Abundance***	***4***
Mammals	***Opossum***	***Species Richness***	***Richness***	***2***
Mammals	***Rat***	***Shannon***	***Compound***	***2***
Mammals	***Rat***	***Shannon Index***	***Compound***	***1***
Mammals	***Rat***	***Simpson***	***Compound***	***1***
Mammals	***Rat***	***Species Abundance***	***Abundance***	***10***

Mammals	*Rat*	*Species richness*	*Richness*	*1*
Mammals	*Rat*	*Species Richness*	*Richness*	*3*
Mammals	*Shrew*	*Shannon*	*Compound*	*4*
Mammals	*Shrew*	*Shannon and Simpson Index*	*Compound*	*2*
Mammals	*Shrew*	*Shannon Index*	*Compound*	*1*
Mammals	*Shrew*	*Species Abundance*	*Abundance*	*17*
Mammals	*Shrew*	*Species Abundance*	*NA*	*1*
Mammals	*Shrew*	*Species Richness*	*Richness*	*7*
Mammals	*Shrew*	*Species Richness*	*NA*	*1*
Mammals	*squirrel*	*Species Richness*	*Richness*	*2*
Mammals	*Squirrel*	*Shannon*	*Compound*	*2*
Mammals	*Squirrel*	*Species Abundance*	*Abundance*	*3*
Mammals	*Vole*	*Activity*	*Abundance*	*2*
Mammals	*Vole*	*Shannon*	*Compound*	*2*
Mammals	*Vole*	*Shannon Index*	*Compound*	*1*
Mammals	*Vole*	*Species Abundance*	*Abundance*	*25*
Mammals	*Vole*	*Species Abundance*	*NA*	*1*
Mammals	*Vole*	*Species Richness*	*Richness*	*9*
Mammals	*Vole*	*Species Richness*	*NA*	*1*
Mammals	*Vole*	*Vole Abudnance Index*	*Abundance*	*1*
Nematodes	*Bacterivores*	*Abundance*	*Abundance*	*99*
Nematodes	*Bacterivores*	*Biomass Carbon*	*Abundance*	*3*
Nematodes	*Bacterivores*	*Enrichment Index*	*Abundance*	*10*
Nematodes	*Bacterivores*	*Relative Abundance*	*Abundance*	*15*
Nematodes	*Entomopathogenic Nematodes*	*DNA Abundance*	*Abundance*	*2*
Nematodes	*Fungivores*	*Abundance*	*Abundance*	*93*
Nematodes	*Fungivores*	*Biomass Carbon*	*Abundance*	*3*
Nematodes	*Fungivores*	*Enrichment Index*	*Abundance*	*10*
Nematodes	*Fungivores*	*Relative Abundance*	*Abundance*	*15*
Nematodes	*General*	*Abundance*	*Abundance*	*109*

Nematodes	***General***	***Biomass Carbon***	***Abundance***	***1***
Nematodes	***General***	***Fisher's Alpha Diversity***	***Compound***	***1***
Nematodes	***General***	***Margalef Taxa Richness***	***Richness***	***3***
Nematodes	***General***	***Margalef Taxonomic Richness***		
Nematodes	***General***	***Shannon***	***Compound***	***58***
Nematodes	***General***	***Shannon trophic Diversity***	***Compound***	***1***
Nematodes	***General***	***Simpson***	***Compound***	***12***
Nematodes	***General***	***Simpson Diversity***	***Compound***	***3***
Nematodes	***General***	***Species Richness***	***Richness***	***3***
Nematodes	***General***	***Taxa richness***	***Richness***	***3***
Nematodes	***General***	***Taxa Richness***	***Richness***	***49***
Nematodes	***Herbivores***	***Abundance***	***Abundance***	***124***
Nematodes	***Herbivores***	***Biomass Carbon***	***Abundance***	***2***
Nematodes	***Herbivores***	***Enrichment Index***	***Abundance***	***10***
Nematodes	***Herbivores***	***Relative Abundance***	***Abundance***	***27***
Nematodes	***Herbivores***	***Shannon***	***Compound***	***1***
Nematodes	***Herbivores***	***Species richness***	***Richness***	***1***
Nematodes	***Omnivores-Predators***	***Abundance***	***Abundance***	***161***
Nematodes	***Omnivores-Predators***	***Biomass Carbon***	***Abundance***	***3***
Nematodes	***Omnivores-Predators***	***Enrichment Index***	***Abundance***	***10***
Nematodes	***Omnivores-Predators***	***Relative Abundance***	***Abundance***	***6***

II-) relative distribution

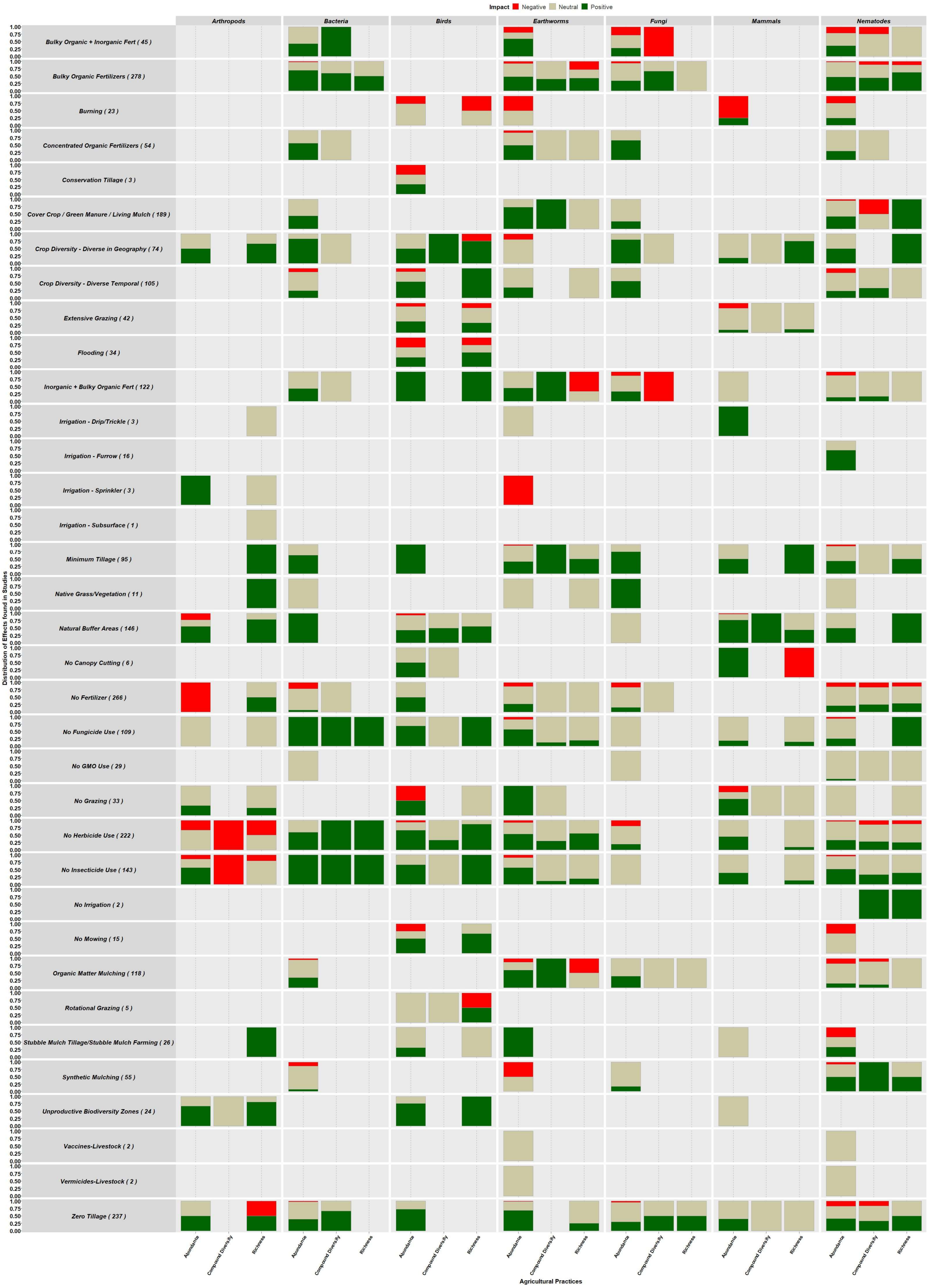

III-) absolute distribution

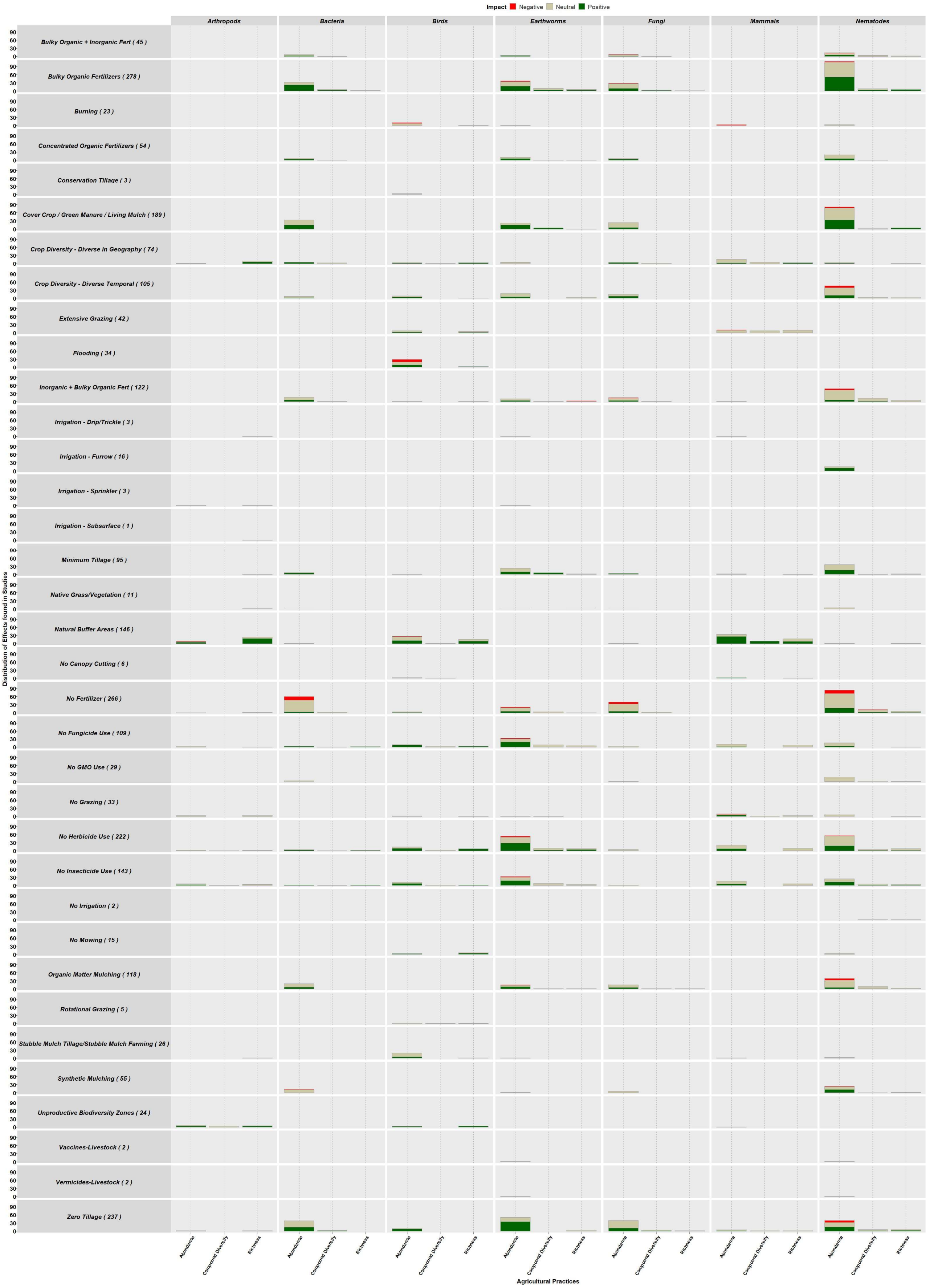

IV-) impact per indicator

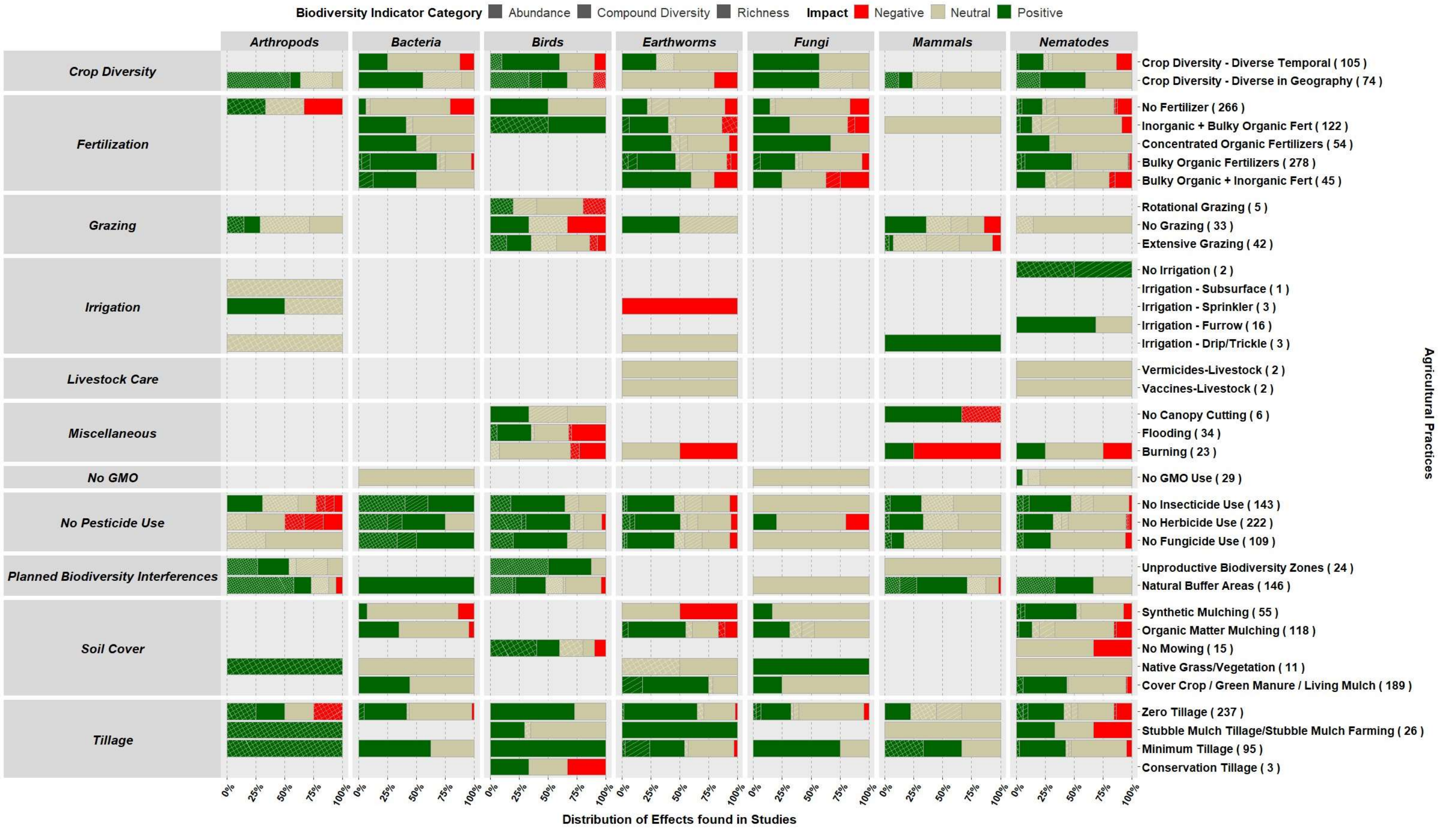

Supplement: Supplementary file 2 — Supplementary Material B [file 44185_2023_34_MOESM2_ESM.pdf]
