# Supplementary material for: Farming practices to enhance biodiversity across biomes: a systematic review
Source: NPJ Biodivers. 2024 Jan 9;3:1. doi: 10.1038/s44185-023-00034-2 (PMC11332212; doi:10.1038/s44185-023-00034-2)
Supplement: Supplementary file 3 — Supplementary Material C [file 44185_2023_34_MOESM3_ESM.pdf]

Group	Taxa	Surrogate Species	SCOPUS	Web Of Knowledge (all databases)	TOTAL post duplicate Removal	Last collection
**Croplands**	*Arthropods*	Bees	TITLE-ABS-KEY (agricult* AND practice AND bee AND (richness OR abundance))	You searched for: TOPIC: (agricult* AND practice AND bee AND (richness OR abundance))	**253**	23/06/2021
		Wasps*	TITLE-ABS-KEY (agricult* AND practice AND wasp AND (richness OR abundance))	You searched for: TOPIC: (agricult* AND practice AND wasp AND (richness OR abundance))	**56**	17/08/2022
	Birds	Birds	TITLE-ABS-KEY (agricult* AND practice AND bird AND (richness OR abundance))	You searched for: TOPIC: (agricult* AND practice AND bird AND (richness or abundance))	**889**	11/12/2021
	Mammals	Small Rodents	TITLE-ABS-KEY (agricult* AND practice AND rodent AND (richness OR abundance))	You searched for: TOPIC: (agricult* AND practice AND rodent AND (richness OR abundance))	**132**	15/03/2022
		Bats	TITLE-ABS-KEY (agricult* AND practice AND bat AND (richness OR abundance))	You searched for: TOPIC: (agricult* AND practice AND bat AND (richness OR abundance))	**62**	15/03/2022
**Grasslands**	*Mammals*	Small Rodents	TITLE-ABS-KEY (((rangeland AND grassland) OR grassland OR rangeland) AND practice AND rodent AND (richness OR abundance))	You searched for: TOPIC: (((rangeland AND grassland) OR grassland OR rangeland) AND practice AND rodent AND (richness OR abundance))	**59**	17/03/2022
		Bats	TITLE-ABS-KEY (((rangeland AND grassland) OR grassland OR rangeland) AND practice AND bat AND (richness OR abundance))	You searched for: TOPIC: (((rangeland AND grassland) OR grassland OR rangeland) AND practice AND bat AND (richness OR abundance))	**14**	17/03/2022
**SOIL**	*Nematodes*	Nematodes	TITLE-ABS-KEY (((agric* AND graz*) OR agric* OR graz*) AND practice AND soil AND nematod* AND (richness OR abundance))	You searched for: TOPIC: (((agric* AND graz*) OR agric* OR graz*) AND practice AND soil AND nematod* AND (richness OR abundance))	**224**	12/02/2022
	Earthworms	Earthworms	TITLE-ABS-KEY (((agric* AND graz*) OR agric* OR graz*) AND practice AND soil AND earthworm AND (richness OR abundance))	You searched for: TOPIC: (((agric* AND graz*) OR agric* OR graz*) AND practice AND soil AND earthworm AND (richness OR abundance))	**250**	12/02/2022
	Fungal/Bacterial Biomass Ratio	Fungal/Bacterial Biomass Ratio	TITLE-ABS-KEY (((agric* AND graz*) OR agric* OR graz*) AND practice AND soil AND bacteri* AND fung* AND ratio AND biomass)	You searched for: TOPIC: (((agric* AND graz*) OR agric* OR graz*) AND practice AND soil AND bacteri* AND fung* AND ratio AND biomass)	**118**	12/02/2022
